# A flexible peptide linking the periplasmic and cytoplasmic domains of MxiG controls type III secretion signaling and stable sorting platform assembly in *Shigella*

**DOI:** 10.3389/fcimb.2025.1611779

**Published:** 2025-08-04

**Authors:** Shoichi Tachiyama, Meena Muthuramalingam, Sean K. Whittier, Yunjie Chang, Jian Yue, Waleed Younis, Wendy L. Picking, Jun Liu, William D. Picking

**Affiliations:** ^1^ Department of Microbial Pathogenesis, Yale School of Medicine, New Haven, CT, United States; ^2^ Microbial Sciences Institute, Yale University, West Haven, CT, United States; ^3^ Department of Pharmaceutical Chemistry, University of Kansas, Lawrence, KS, United States; ^4^ Department of Veterinary Pathobiology, University of Missouri, Columbia, MO, United States; ^5^ Christopher S. Bond Life Sciences Center, University of Missouri, Columbia, MO, United States; ^6^ Department of Microbiology, Faculty of Veterinary Medicine, South Valley University, Qena, Egypt

**Keywords:** Shigella, type III secretion system, injectisome, MxiG, sorting platform

## Abstract

*Shigella flexneri* uses its type III secretion system (T3SS) to invade human enterocytes. The T3SS injectisome is controlled by proteins at the tip of an exposed needle that sense host cell contact. Substrate selection and powering of secretion is controlled by a cytoplasmic assembly called the sorting platform (SP). The *Shigella* SP possesses six pod structures linked to a central ATPase via radial spokes. The SP associates with the injectisome inner membrane ring (IR) via the adaptor protein MxiK. The major IR component is MxiG, whose globular periplasmic domain (MxiG^P^) packs with MxiJ in a 24-fold symmetry. MxiG also has a transmembrane helix attached to a small cytoplasmic domain (MxiG^C^) via a flexible linker peptide. Change from the IR’s 24-fold symmetry to six-fold symmetry for the SP in *Shigella* occurs via MxiG^C^ pairs that associate with MxiK. The intervening pairs shift to the center of the IR/SP assembly, which is distinct from what is seen for *Salmonella*. This implicates the linker in dynamic motions at the IR-SP interface, but the functional importance of the linker is unknown. Using a library of mutants, we found that the linker can accept diverse mutations without eliminating injectisome function. However, some mutants were found to give rise to subpopulations able to form needles and secrete effectors in the absence of a stably assembled SP. Mutants lacking the entire linker could not secrete any effector proteins (e.g. the IpaD tip protein) and had no T3SS-related virulence functions, however, there were subpopulations that could still secrete MxiH and assemble visible needles. In contrast, a very short linker could export IpaD to the needle tip, but could not rapidly respond to external secretion signals and were thus unable to quickly enter epithelial cells. These findings implicate the MxiG linker in signaling processes that are sensed at the needle tip. Our findings suggest that the native MxiG linker peptide has evolved to maximize T3SS function at steps beyond needle formation, while needle formation can occur even when the SP is highly destabilized.

## Introduction

*Shigella flexneri* is a Gram-negative bacterial pathogen that causes bacillary dysentery (shigellosis) (Kotloff et al., 2018). Onset of infection occurs when the bacteria are transcytosed across M cells in the colon to reside in the *lamina propria* where they invade and kill macrophages to gain access to the colonic submucosa (Ranganathan et al., 2019). *Shigella* then invades the overlying epithelial cells, escapes into the cytoplasm where it replicates, and then directly enters adjacent colonocytes using actin-based motility (Mattock and Blocker, 2017). *Shigella* invasion requires a type III secretion system (T3SS) to inject host altering effector proteins into target cells (Muthuramalingam et al., 2021). In macrophages, these effectors cause cell death by pyroptosis (Guichon et al., 2001), but in epithelial cells they elicit the formation of membrane ruffles that ultimately enclose *Shigella* in a large vacuole (Killackey et al., 2016). After vacuolar escape, the pathogen replicates and uses IscA to promote actin-based motility in a T3SS-independent manner (Bernardini et al., 1989), followed by spread and escape into adjacent cells with contributions from the T3SS translocators/effectors IpaB and IpaC (Duncan-Lowey et al., 2020).

The T3SS apparatus, or injectisome, visually resembles a needle and syringe with four subassemblies. An exposed needle tip complex contacts the host cell membrane to rapidly induce secretion, thereby initiating formation of a translocon pore through which effector proteins are delivered to the host cytoplasm (Espina et al., 2006; Olive et al., 2007). This is enabled by an extracellular needle that serves as the conduit for effector delivery (Deane et al., 2006). The needle is anchored to an envelope-spanning basal body formed by inner and outer membrane rings with an inner rod connected to the export apparatus and cytoplasmic export gate (Blocker et al., 1999; Sekiya et al., 2001). The whole secretion process is then controlled by a cytoplasmic sorting platform (SP) that is involved in selecting effector proteins and providing energy for protein translocation (Hu et al., 2015; Soto et al., 2022). The dominant protein densities within the SP are the hexameric Spa47 (SctN) ATPase and six evenly spaced pod structures (for injectisome organization, see **Supplementary Figure S1** and the unified Sct nomenclature for virulence T3SS is provided in **Supplementary Table S1**) (Hu et al., 2015). The Spa47 ATPase is believed to separate selected effector proteins from their chaperones so that the unfolded effectors can be delivered to the export gate and translocated into the host cell (Jouihri et al., 2003; Burgess et al., 2016). Spa47 is held in place by MxiN (SctL) which forms six radial spokes connecting the ATPase to six large pod structures (Jouihri et al., 2003; Case and Dickenson, 2018). Each pod appears to contain at least one copy of a heterotrimer consisting of one full-length copy of Spa33 (SctQ) and two copies of a C-terminal Spa33 SPOA2 domain fragment that is generated via an alternative translation start site within *spa33* (Notti et al., 2015; McDowell et al., 2016; Tachiyama et al., 2021), Each pod associates with MxiK (SctK), which is the adaptor protein connecting the SP assembly to the inner-membrane ring (IR) protein MxiG (SctD) via its small cytoplasmic domain referred to here as MxiG^C^ (SctD^C^) (Tachiyama et al., 2019; Muthuramalingam et al., 2020).

MxiG possesses two independently folding globular domains. The C-terminal portion resides in the periplasm (designated here as MxiG^P^/SctD^P^) and the cytoplasmic N-terminal domain (MxiG^C^) associates with MxiK to form the IR-SP interface (McDowell et al., 2011; Muthuramalingam et al., 2020). MxiG^C^ and MxiG^P^ are bridged by a transmembrane helix (TMH) and a flexible cytoplasmic linker that tethers the TMH to MxiG^C^. The crystal structure of MxiG^C^ reveals a noncanonical forkhead-associated (FHA) that we have shown directly interacts with MxiK (Tachiyama et al., 2019). Unfortunately, no high-resolution structure is available for MxiK (McDowell et al., 2011), however, the orientation of MxiK within the SP was demonstrated using insertional mutagenesis to show that the C-terminus is located within the “cage” structure of the SP (Tachiyama et al., 2019). The crystal structure of the MxiK homologue from *Pseudomonas aeruginosa* (PscK/SctK) has been solved and found to readily fit within the MxiK density of the *Shigella* injectisome with the MxiK-MxiG^C^ interface defined roughly based on mutagenesis studies (Muthuramalingam et al., 2020).

The cytoplasmic domain of the *Salmonella* MxiG homologue PrgH/SctD (referred to here as PrgH^C^/SctD^C^) was reported to form six distinct clusters of four at the point where they contact the MxiK homologue OrgA (Hu et al., 2017; Soto et al., 2022). This is in sharp contrast with the PrgH cytoplasmic domain’s position in purified T3SS needle complexes (NCs) which lose the SP during their extraction. *Salmonella* NCs have an evenly spaced 24-fold symmetrical ring of PrgH^C^ that is shifted inward (Marlovits et al., 2004). Likewise, MxiG^C^ appears to show that this domain is uniformly shifted inward when viewed *in situ* for *mxiK* null mutants (Hodgkinson et al., 2009). These *in situ* and *in vitro* structures from the *Shigella* and *Salmonella* injectisomes, respectively, indicate that the interface between the IR and SP is a dynamic region and this may be mechanistically important for injectisome function. Dynamic movements within this region are anticipated to be facilitated via the MxiG/PrgH linker that connects MxiG^C^/PrgH^C^ with the MxiG/PrgH TMH. This flexibility may have evolved in a T3SS-specific manner to optimize the coordinated protein-protein interactions needed to maximize secretion efficiency. The *Salmonella* (PrgH) and *Shigella* (MxiG) linkers differ at the sequence level and in length (**Supplementary Figure S2**), so the question arises as to how linker features affect SP assembly and secretion efficiency in these systems.

Here we altered the content, flexibility and length of the MxiG linker peptide to determine how linker composition contributes to SP assembly and injectisome function. While many of the introduced mutations had relatively little effect on injectisome function, injectisomes with MxiG completely lacking or having a very short linker did show a loss of virulence functions. To look more closely at the impact of selected mutations on injectisome assembly, we used cryo-electron tomography (cryo-ET) and sub-tomogram averaging to determine *in situ* injectisome structures using *Shigella* minicells. The resulting data revealed surprising effects of certain MxiG linker mutations on SP assembly and the injectisome’s ability to form extracellular needles.

## Results

### *In situ* structure of the *Shigella* injectisome reveals the MxiG^C^ and MxiK interface

**Figure 1:**
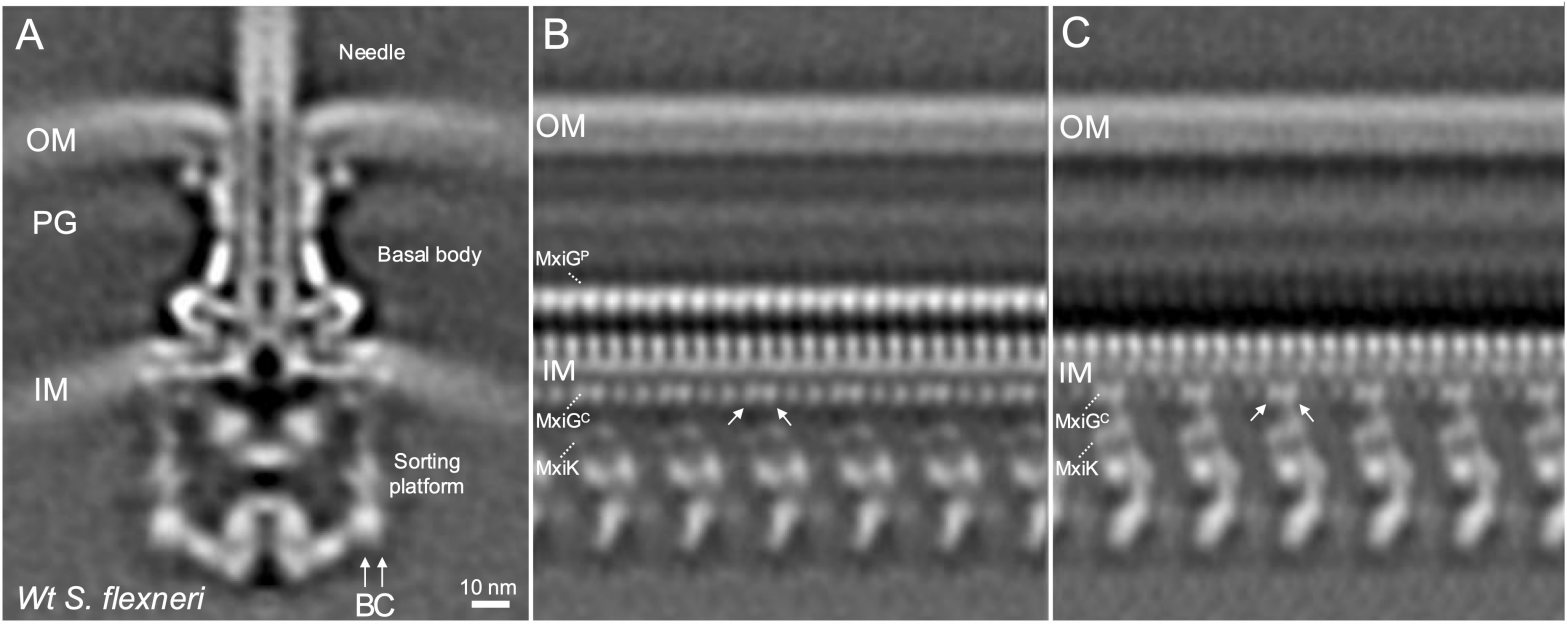
The cytoplasmic portion of the IR ring has 24-fold symmetry with pairs of MxiG^C^ associated with MxiK and the intervening pair shifted toward the center of the SP. **(A)** shows the overall structure of the wildtype *Shigella* T3SS injectisome. The SP is at the bottom with the basal body visible between the outer membrane (OM) and inner membrane (IM). The peptidoglycan cell wall is indicated by PG. The extracellular needle is visible at the top. **(B)** shows an unfolded side view of the injectisome at the position of arrow B in Panel A. From this view, the main densities of the Spa33 pods are visible at the bottom and 24 copies of MxiG^C^ are visible just below the IM and 24 copies of MxiG^P^ are visible within the periplasm. Also visible are the cell wall and the OM. **(C)** shows an unfolded side view at the position of arrow C in Panel A. In this view, the MxiK adaptor is seen associated with two copies each of MxiG^C^, however the other 12 MxiG^C^ densities are not visible in this view.

To investigate the interface between MxiG^C^ (IR) and MxiK (SP adaptor protein), a local refinement was performed for the *in situ* structure of *Shigella* injectisome (**Figure 1**) with C1 symmetry as part of a reanalysis of findings we previously reported (Tachiyama et al., 2019). The unrolled view at the inward cross section of the MxiG^C^ and MxiK interface (vertical cross-section at arrow B in **Figure 1A**) shows 24 copies of MxiG^C^ (**Figure 1B**). MxiK is poorly visible at this section though a portion of the pods (Spa33) can be seen. When the cross section is moved outward (arrow C in **Figure 1A**), one pair of MxiG^C^ is seen associated with MxiK, which is now clearly visible, with the intervening MxiG^C^ pair displaying a weak density (**Figure 1C**). This *in situ* structural analysis indicates that the MxiG^C^ position is flexible to accommodate interactions with MxiK of the SP, with the alternating pairs of MxiG^C^ shifted inward toward the core of the SP during the protein translocation (**Supplementary Figure S3**). Furthermore, this is distinct from what was reported for PrgH^C^ in *Salmonella*, where this domain forms six clusters of four at the point where they form an interface with OrgA (Hu et al., 2017; Soto et al., 2022).

### Contact-mediated hemolysis of MxiG linker mutants

**Figure 2:**
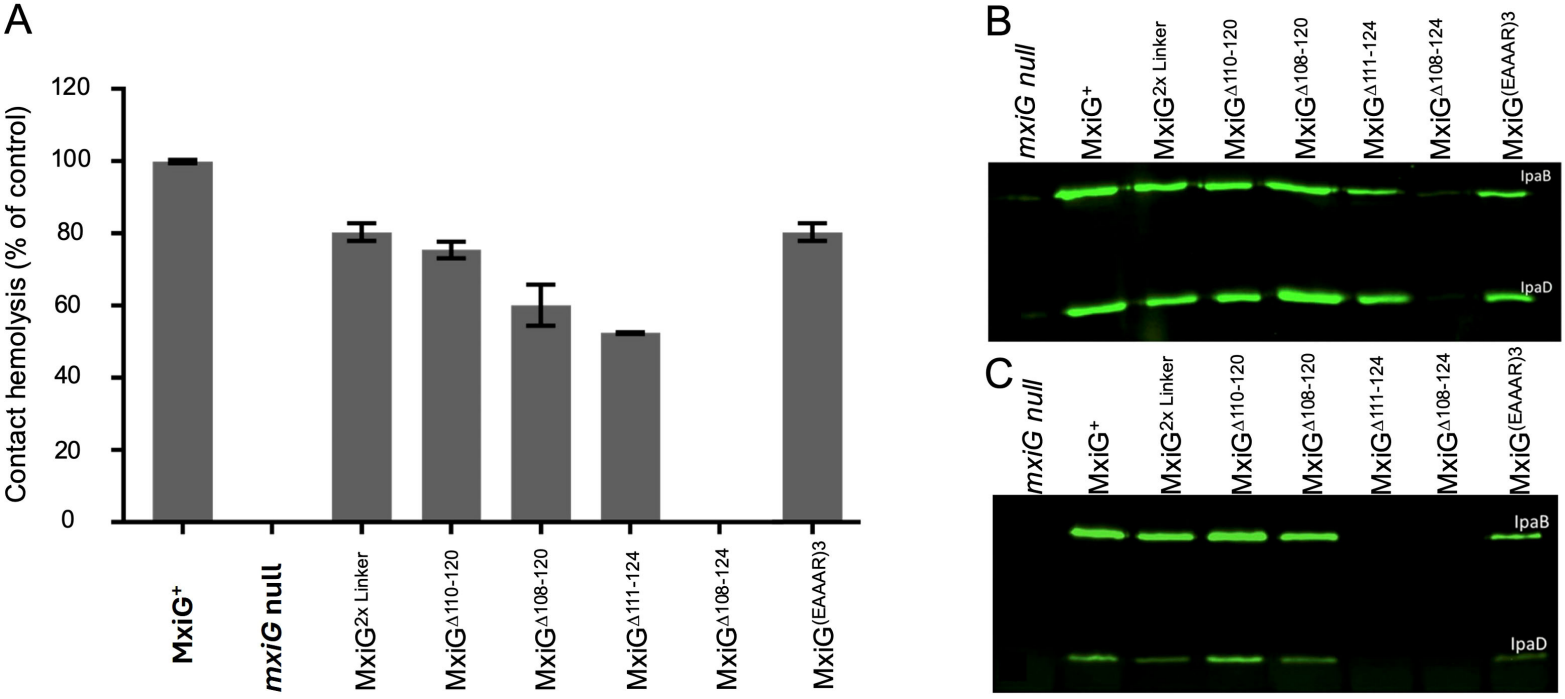
The activity of *Shigella* expressing selected MxiG linker mutants are shown with respect to contact-mediated hemolysis and Ipa protein secretion. **(A)** shows the relative hemolysis activities in *Shigella* as described in Methods. All of these *Shgella* strains show some level of hemolysis activity except for the *mxiG* null strain and the MxiG^Δ108–124^ strain. The hemolysis data presented here are from three technical replicates for each strain (average ± SD) and is representative data of two independent experiments. **(B)** shows an immunoblot for these same strains for the supernatants of overnight cultures using mouse serum containing anti-IpaB and anti-IpaD IgG. The overnight steady-state secretion profile parallels the hemolysis profile seen in panel **(A) (C)** shows the rapid secretion of IpaB and IpaD induced following incubation with CR. In this case, the bacteria were harvested, suspended in buffered saline and allowed to secrete for 20 min in the presence of CR. These immunoblots were performed at least twice and the blots presented here are representative of what was observed each time.

To determine how changes in the MxiG linker peptide affect injectisome function, contact hemolysis was initially examined. Contact-mediated hemolysis provides a highly reproducible and convenient measure of whether *Shigella* can secrete Ipa proteins to the tip complex to assemble functional translocons following forced target cell contact (Sansonetti et al., 1986; Picking et al., 2005). A summary of all the mutations introduced into the linker is provided in **Supplementary Table S2**. The remainder of this report will focus on a subset of these mutants. Most amino acid substitutions, small insertions and small deletions had negligible effect on hemolysis (**Supplementary Table S2**). Likewise, replacement of the entire linker with that of PrgH from *Salmonella* had no detectable effect on translocon formation. We therefore tested hemolysis for progressively larger deletions and observed a corresponding reduction in hemolysis until the linker was completely eliminated (MxiG^Δ108-124^), at which point there was no detectable contact-mediated hemolysis (**Figure 2A**). We also examined hemolysis when two tandem linkers were used to replace the native single linker of MxiG (MxiG^2×Linker^) and found it continued to display 80% of the hemolysis activity seen for the wildtype linker (**Figure 2A**), suggesting that once the linker has reached a length that can accommodate adequate movement for making contact with MxiK, then additional length/flexibility has a relatively minor effect on injectisome function. Furthermore, because the Δ108–120 and Δ111–124 linkers were both at least 50% active, it appears that as few as three amino acids is sufficient to form a linker capable of supporting a functional, albeit suboptimal, injectisome and this is not sequence dependent. Furthermore, an inactive form of bacteriophage T4 lysozyme (T4L) (Barta et al., 2018) could be introduced at the center of the wildtype linker or between two copies of the wildtype linker with only 60 and 70% loss of contact hemolysis activity, respectively (**Supplementary Table S2**). This was not the case, however, for green fluorescent protein (GFP) inserted at the center of the linker, which had no detectable activity (**Supplementary Table S2**).

To determine the importance of linker flexibility for injectisome function, four different mutants in which the linker was replaced with poly-proline segments of 15 to 18 residues in length were generated, based on the use of poly-proline as a rigid molecular ruler (Moradi et al., 2009). These mutants still restored 60, 63, 54 and 59% of wildtype MxiG hemolysis activity, respectively (**Supplementary Table S2**). Thus, linker flexibility contributes to, but is not a strict requirement for, injectisome function. Notably, these rigid mutant linkers can adopt a left-handed PPII helical structure that has a degree of flexibility that would allow some movement of the MxiG^C^ FHA domain. As an alternative to poly-proline, we introduced consecutive EAAAR sequences in place the native MxiG linker. This sequence is a strong α-helix former (Merutka et al., 1991) and would give a progressively longer and highly rigid α-helical structures that would be more constrained at each end (**Supplementaryry Figure S4**). Two and three EAAAR replacements resulted in 4 and 73% hemolytic activity, respectively (**Figure 2A** and **Supplementary Table S2**). These data suggest that linker rigidity is acceptable, but only when the length is beyond a critical point if linker flexibility is further constrained. Based on the contact-mediated hemolysis results, we selected a subset of linker variants to be considered further, including the double tandem linker (2×Linker), the four large deletion mutant linkers (including MxiG^Δ108-124^), and the MxiG^(EAAAR)3^ linker (see **Figure 2**). The contact-mediated hemolysis results for the selected mutants are shown in **Figure 2A**.

### Secretion of Ipa proteins

Contact-mediated hemolysis provides a measure of translocon formation; however, it is not a substitute for directly examining Ipa protein secretion into *Shigella* culture supernatants for the selected linker mutants. *Shigella* type III secretion can be monitored by detecting Ipa protein secretion into the extracellular milieu of overnight cultures using immunoblot analysis. We used this method to detect IpaB and IpaD in overnight culture supernatants for the selected linker mutants (**Figure 2B**) and found that all of the mutants tested, including the double tandem mutant (2×Linker), MxiG^Δ110-120^, and MxiG^Δ108–120^ secreted IpaB and IpaD at levels comparable to that of *Shigella* expressing wildtype MxiG (**Figure 2B**). *Shigella* expressing MxiG^Δ111–124^ and MxiG^(EAAAR)3^ also secreted IpaB and IpaD, but at reduced levels. Meanwhile, the ΔLinker mutant (MxiG^Δ108-124^) failed to secrete any detectable levels of IpaB or IpaD. Quantification of the overnight secretion of IpaB for the majority of the MxiG linker mutants is provided in **Supplementary Figure S5**. The only two mutants completely incapable of overnight secretion were the MxiG^Δ108–124^ mutant and the mutant with GFP inserted into the linker (**Supplementary Figure S5**). Quantification of overnight IpaB secretion for the immunoblot presented in **Figure 2B** is shown in **Supplementary Figure S6** (left side), which clearly shows that MxiG^Δ111–124^ is reduced in secretion relative to the wildtype MxiG and the other deletion mutants.

The overnight secretion data largely parallel the contact-mediated hemolysis data except for the reduced secretion by the MxiG^Δ111–124^ and MxiG^(EAAAR)3^ mutants (**Figure 2B**). This prompted us to consider the effects these mutations might have on the rapid induction of secretion observed when *Shigella* is incubated for a short time with Congo red (CR) (Espina et al., 2006). In this assay, the *Shigella* are collected by centrifugation, washed, resuspended in PBS and then incubated with CR for 20 min. The IpaB and IpaD present in the resulting supernatants were detected by immunoblot analysis. The results of induced secretion were similar to those seen for overnight secretion for most of the selected MxiG mutants except that in this case both the MxiG^Δ108–124^ and MxiG^Δ111–124^ failed to show any detectable levels of IpaB or IpaD secretion (**Figure 2C**). MxiG^Δ111–124^ is the first *Shigella* strain observed in our laboratory that has been found to be positive for contact-mediated hemolysis while unable to display CR-induced Ipa protein secretion, perhaps indicating dysfunction in the transmission of environmental signals to the SP for this mutant. The implication is that not only are the needle and tip complex involved in signaling for induced secretion, but MxiG of the IR also plays a role in responding to the environmental signals for secretion via its linker peptide. Quantification of IpaB secretion following CR induction is shown in **Supplementary Figure S6** (right side), which clearly shows that MxiG^Δ108–124^ and MxiG^Δ111–124^ are both unable to respond to rapid secretion induced by CR.

### *Shigella* invasion of HeLa cells

**Figure 3:**
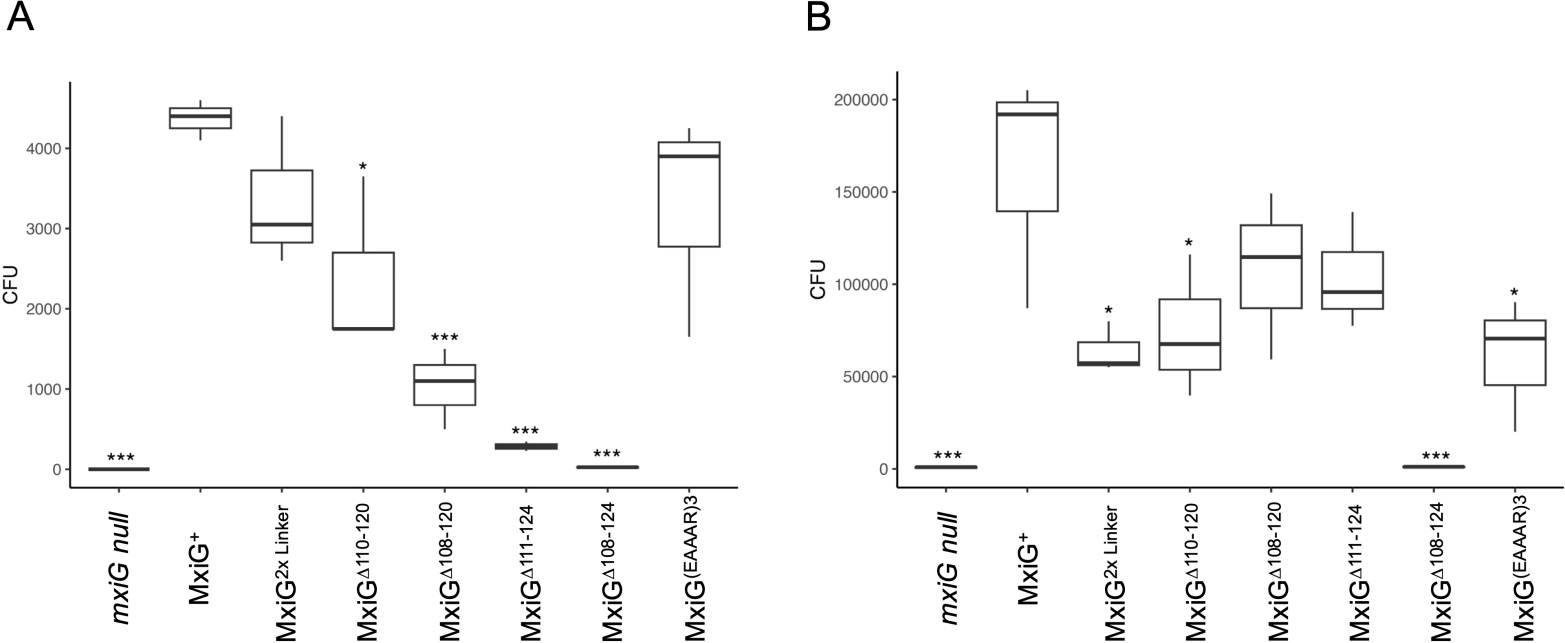
*Shigella* invasion of epithelial cells was determined using HeLa cells. **(A)** shows the relative invasion then the bacteria are added to HeLa cell monolayers, agitated and then allowed to incubate for 30 minutes prior to aspiration, washing and adding a gentamycin solution. **(B)** is the same assay except that the bacteria were centrifuged to force contact with the HeLa cells and the incubation was for 1 h. The overall higher invasion in Panel B is due to the forced contact and longer incubation time. These invasion assays were carried out in triplicate for each strain. The findings were verified in at least two independent trials. * p < 0.05, *** p < 0.0005.

Traditional contact-mediated hemolysis by *Shigella* involves forced contact between *Shigella* and erythrocytes followed by an extended (≥1 h) incubation. Likewise, overnight secretion is reflective of a low-level steady state-secretion that occurs over a prolonged time. In contrast, CR-induced secretion is a rapid onset of secretion as might occur upon *Shigella* contact with host cells. We therefore assessed each of the selected mutant’s ability to invade cultured HeLa cells using two variations of a standard gentamycin protection assay (Picking et al., 2005). Initially, the bacteria were incubated with HeLa cell monolayers without forced contact over a 30 min incubation time. In this case, the *mxiG* null strain and the MxiG^Δ108–124^ showed no ability to invade the HeLa cells as was expected (**Figure 3A**). Interestingly, the MxiG^Δ111–124^ mutant was also unable to restore any significant invasiveness, which parallels the results for CR-inducted secretion (**Figure 2C**). As importantly, the MxiG^Δ108–120^ mutant was significantly reduced in its ability to invade, indicating that these very short linkers are functionally compromised. In contrast, the 2×Linker and MxiG^(EAAAR)3^ mutants invaded at levels that were not statistically different from that of wildtype MxiG (**Figure 3A**). The MxiG^Δ110–120^ mutant was also reduced for rapid invasion (p< 0.05), which further suggests that linker length has a role in this secretion efficiency and virulence functions. These data imply that rapid secretion induction is required for optimal invasion of host cells.

Unlike the invasion assay described above, standard invasion assays with *Shigella* often include a centrifugation step to force bacterial contact with the cell monolayers (Picking et al., 2005). We therefore performed the invasion assay with HeLa cells in which the bacteria were centrifuged onto the monolayer and with an extended (1 h) incubation time prior to the addition of gentamycin (**Figure 3B**). This mimics the conditions used in the standard contact-mediated hemolysis assays and the overnight secretion analyses (**Figures 2A, B**). When these conditions are used, the only MxiG mutant unable to show substantial invasion is the MxiG^Δ108-124^. In this case the MxiG^Δ111–124^ mutant’s invasion potential is different from, but not considered statistically different from, wildtype MxiG (p > 0.05) (**Figure 3B**). Collectively, these data show that the MxiG linker has a major role in injectisome function, and this may be related to its central role in the rapid mobilization of Ipa protein secretion in response to extracellular signals.

### Surface expression of IpaD

A nascent *Shigella* injectisome’s external needle is immediately capped with a tip complex that is initially composed of a pentamer of IpaD (Epler et al., 2012). To determine the importance of IpaD surface presentation on downstream events, we used flow cytometry to determine the presence of IpaD on the surface of *Shigella* expressing the different MxiG forms generated here. The raw flow cytometry data for key mutants are presented in **Supplementary Figure S7** and the quantification of IpaD on the *Shigella* surface are presented in **Supplementary Table S2**. It is interesting to note that only the *mxiG* null *Shigella* strain and *Shigella* expressing the MxiG mutants MxiG^Δ108-124^, GFP at position 115, and MxiG^(EAAAR)2^ fail to display IpaD on their surfaces and these are the mutants that are defective in all of the aspects of injectisome function tested here (MxiG^(EAAAR)2^ was not followed up further in this study). Even the mutants lacking the rapid secretion phenotype have significant levels of IpaD present at the bacterial surface. This may not be surprising because this process is one that occurs over time (during injectisome assembly) and not one that is a result of rapid T3SS induction.

### *In situ* visualization of the *Shigella* injectisomes in three linker mutants

**Figure 4:**
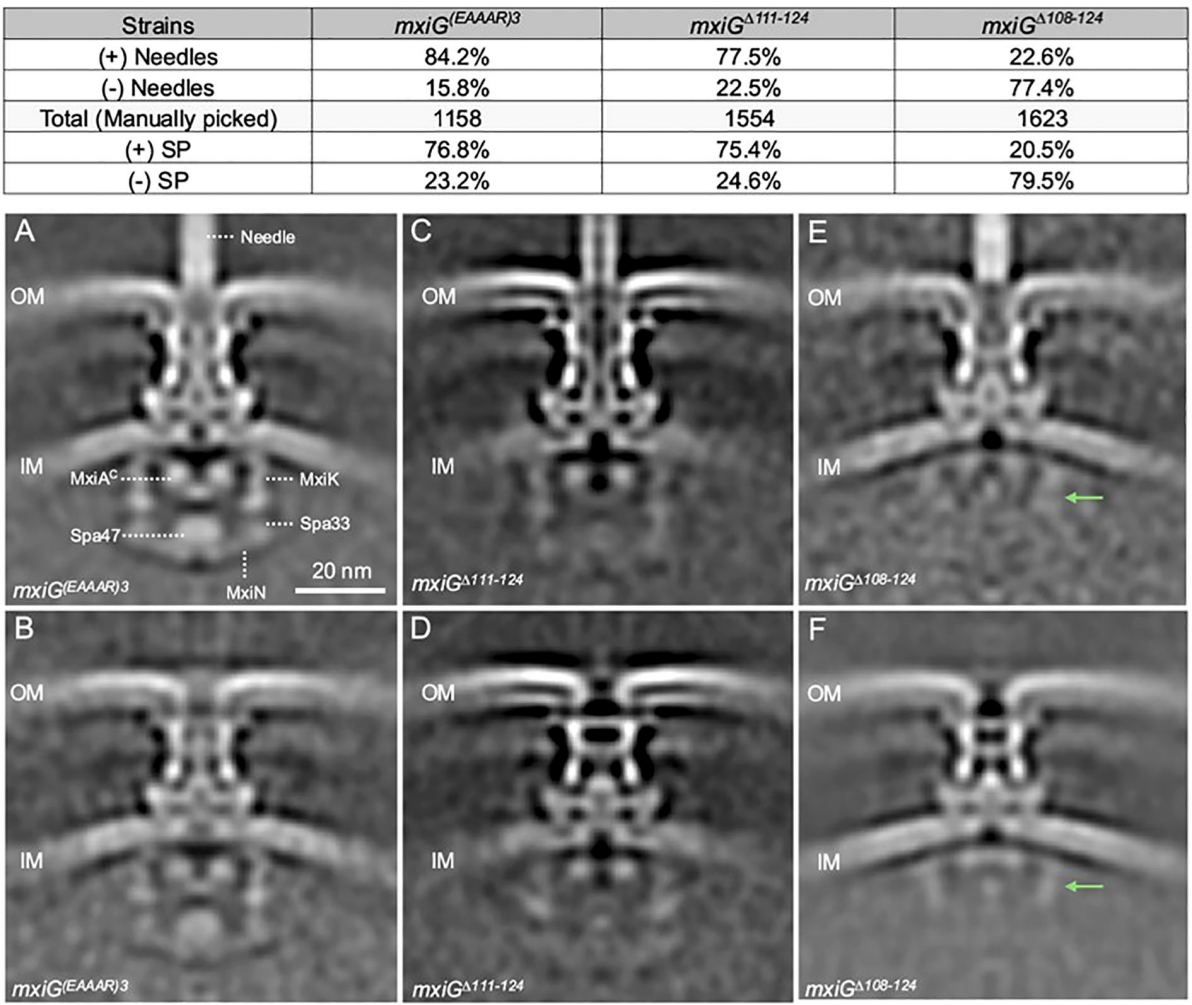
Cryo-ET analysis was used to determine the injectisome structure for MxiG^(EAAAR)3^, MxiG^Δ111-124^, and MxiG^Δ108-124^. Visualization of the specimens indicated two injectisome populations in each case (including for the *Shigella* expressing wildtype MxiG). The Top Panel shows the relative abundance of injectisomes in each case either possessing or lacking visible needles formed by secreted MxiH. Sub-tomagram averaging allowed for determining the structure of each mutant population. **(A, C, E)** show the structures for injectisome populations possessing needles for MxiG^(EAAAR)3^, MxiG^Δ111-124^, and MxiG^Δ108-124^, respectively. **(B, D, F)** show the structures for injectisome populations lacking needles for MxiG^(EAAAR)3^, MxiG^Δ111-124^, and MxiG^Δ108-124^, respectively. The green arrows in the panels **(E, F)** indicate MxiK and top part of Spa33 position in the SP. The population of the SP assembly in each mutant was determined by the 3D classification for the averaged structures and showing in the top panel.

Based on the data presented above, we selected three linker mutants having distinct phenotypes for in-depth cryo-ET analysis. To investigate injectisomes from the MxiG^Δ108-124^, MxiG^Δ111-124^, and MxiG^(EAAAR)3^ mutants, purified minicells making each of these mutants were visualized by cryo-ET. Interestingly, injectisomes from all three mutants and even wildtype *Shigella* could be separated into two major structural classes, injectisomes possessing a visible extracellular needle and those lacking visible needles (see **Supplementary Figure S8**). This is particularly interesting for the MxiG^Δ108–124^ mutant because it lacks any surface presentation of IpaD while the MxiG^(EAAAR)3^ and MxiG^Δ111–124^ mutants display IpaD on the surface at levels comparable to the wildtype MxiG (**Supplementary Figure S7**; **Supplementary Table S2**). Thus, injectisomes on minicells in tomograms were manually selected to examine these two classes (those possessing and those lacking visible needles). Approximately 84% of injectisomes from the minicells making MxiG^(EAAAR)3^ had visible needles assembled on their surface and 78% of minicells making MxiG^Δ111–124^ had needles on their surface, while in each case the remainder of the injectisomes lacked any visible needles (**Figure 4**, top). In contrast, less than a quarter of the injectisomes from the MxiG^Δ108–124^ mutant minicells possessed visible needles (**Figure 4**, top), even though this mutant had no detectable contact hemolysis activity (**Figure 2A**), did not possess any detectable IpaD on the surface (**Supplementary Figure S7**), and did not secrete any IpaD or IpaB (**Figures 2B, C**). These data show that injectisomes expressing MxiG lacking any linker, like MxiG^Δ108-124^, can translocate the needle protein monomer MxiH, which can then assemble into recognizable needles on the bacterial surface (**Supplementary Figure S8**).

### *In situ* structures of injectisomes from three MxiG mutants

To better understand the overall structural features for the two injectisome classes, we more closely examined the *in situ* structures of injectisomes for *Shigella* making MxiG^(EAAAR)3^, MxiG^Δ111-124^, and MxiG^Δ108–124^ using sub-tomogram averaging (**Figure 4**; **Supplementary Table S3**). For the MxiG^(EAAAR)3^ mutant, the injectisomes possessing needles had a clearly defined and organized SP with densities visible for MxiK (adaptor protein), Spa33 (pod structures), MxiN (radial spoke protein) and Spa47 (ATPase) (**Figure 4A**). There was also a well-defined export gate (MxiA) assembly (**Figure 4A**). The structural features of the SP in this mutant are almost identical to those of the wildtype injectisome (see **Figure 1A**). For the MxiG^(EAAAR)3^ mutants lacking visible needles, a similar SP can be seen (**Figure 4B**). Meanwhile, MxiG^Δ111–124^ retains most basic injectisome functions (IpaD surface presentation, contact hemolysis and invasion of HeLa cells), however, it possesses a SP with less readily identified densities at the positions normally occupied by the adaptor (MxiK), pods (Spa33), radial spokes (MxiN), and ATPase (Spa47), regardless of whether or not needles are present (**Figures 4C, D**). Despite this, the basal body is well defined and the export gate (MxiA), especially for the injectisomes with needles (**Figure 4C**), is well defined and positioned much like what is seen for the MxiG^(EAAAR)3^ mutant (**Figure 4A**) and the wildtype injectisome (**Figure 1A**). It is worth noting that for the MxiG^Δ111–124^ mutant, the basal body seems to adopt a “closed” state for the injectisomes lacking needles, meaning that it lacks a continuous central channel through the periplasm (**Figure 4D**).

In contrast to injectisomes for the MxiG^(EAAAR)3^ and MxiG^Δ111–124^ mutants, the *in situ* structure of injectisomes for the MxiG^Δ108–124^ mutant does not possess a clear and fully intact SP, whether needles are visible or not (**Figures 4E, F**). In the cytoplasmic region of these injectisomes, weak densities are observed at the positions of the adaptor and top portion of the pods, however, the bottom portion of the pods, the radial spokes and the ATPase are not visible in these SP assemblies (**Figures 4E, F**). Moreover, the basal body in the absence of needles has an apparent “closed” structure while that in the presence of needles appears to be a hybrid between “open” and “closed” (**Figures 4E, F**). These data suggest that the SP is highly destabilized when the MxiG linker is removed. Nevertheless, secretion of at least “early substrates” (*i.e.* MxiH) does not appear to require either a complete or a stable SP assembly (**Figure 4**).

To further explore SP assembly in these mutants, a second structural classification was performed to separate subtomograms into two populations based on the presence or absence of SP densities rather than needle assembly. Using this analysis, we found that approximately 80% of the MxiG^(EAAAR)3^ and MxiG^Δ111–124^ injectisomes possessed some level of SP assembly (**Figure 4**, top and **Supplementary Figure S9**). In contrast, for the MxiG^Δ108–124^ mutant only about 20% of the injectisomes possessed densities that could be attributed to the SP (**Figure 4**, top and **Supplementary Figure S9**), and these densities were mostly limited to the upper portions of the SP (adaptor protein and top portion of the pods). The densities of the bottom portion of the pods, the radial spokes, and the ATPase are less or differently organized when compared to the same regions of the SPs from wildtype MxiG and MxiG^(EAAAR)3^ injectisomes (**Figures 1A**, **4A**, and **Supplementary Figure S9**). Some SP density is also observed in the averaged structures of injectisomes for which the SP was not seen in individual subtomograms (**Supplementary Figure S9**). This density is most clear for upper portions of the SP pods (**Supplementary Figures S9B, S9F**), but faint density is observed for lower portions as well (**Supplementary Figure S9D**). These findings show that MxiG without the linker is still able to assemble some portions of the SP, but less efficiently for the lower regions of the SP.

Interestingly, when injectisomes are classified based upon the presence or absence of SP densities, populations were present in each class that possessed visible needle densities (**Supplementary Figure S9**). Examination of the data from the two separate injectisome classifications shows that the presence/absence of the needle and the SP in individual subtomograms are uncorrelated (**Supplementary Table S4**). In the case of the MxiG^(EAAAR)3^ mutant, for example, we observe that nearly 69% of injectisomes have both SP and extracellular needle density, which is close to the expected frequency of 65% if the presence of SP density in an injectisome is independent of the presence of a needle. This independence holds for the double-negative state (no SP and no needle) as well as each single-negative state, and for each mutant in **Figure 4**. The observed frequencies differ slightly from expected of full independence, but this is likely due to the fact that it is more difficult to accurately classify individual injectisomes based on the presence/absence of the SP rather than needles. In contrast, when cryo-ET was used to examine injectisomes from *Shigella* minicells expressing native MxiG along with an intact, but catalytically inactive, *spa47* ATPase mutant (Burgess et al., 2016), there were no needles present on any of the injectisomes despite there being a fully intact and organized SP in the averaged structure (**Supplementary Figure S10**). These ATPase mutants also possessed a basal body that was in what we’ve termed a “closed” state with regard to the presence of a central channel through the periplasm. The fact that a class of MxiG^Δ108–124^ mutants is able to form needles with a highly destabilized SP (see **Figures 4E, F**, and **Supplementary Figure S9F**) suggests that a transiently assembled SP is sufficient for MxiH secretion, though not for secretion of subsequent T3SS substrates (*e.g.* IpaD). Furthermore, despite not being able to secrete IpaD, the lengths of the needles formed for the MxiG^Δ108–124^ mutant were comparable to the length of the needles observed for the other MxiG mutants and wildtype MxiG (see **Supplementary Figure S4C**).

One final observation for the MxiG^Δ108–124^ mutant was that the density of the cytoplasmic portion of the export gate (MxiA^C^ in **Figure 4A**) was visible but shifted upward when the injectisome class lacking a visible SP was examined (**Figure 4F**, **Supplementary Figure S9F**). This movement was less pronounced for the other injectisomes lacking a visible SP (**Supplementary Figures S9B, D**) and this was not observed for any of the injectisomes possessing a visible SP (**Supplementary Figures S9A, C, E**). When examining injectisomes classified by the presence or absence of needles, both populations for MxiG^Δ108–124^ had the export gate shifted upward (**Figures 4E, F**). These findings suggest there is communication between the SP and the export gate. Moreover, absence of an upward shift for MxiA^C^ by the MxiG^Δ111–124^ and MxiG^(EAAAR)3^ mutants lacking visible SP components suggests that this communication still occurs, most likely because of transient SP interactions. The molecular basis for communication between the SP and export gate is unclear but may be of importance for future studies.

## Discussion

**Figure 5:**
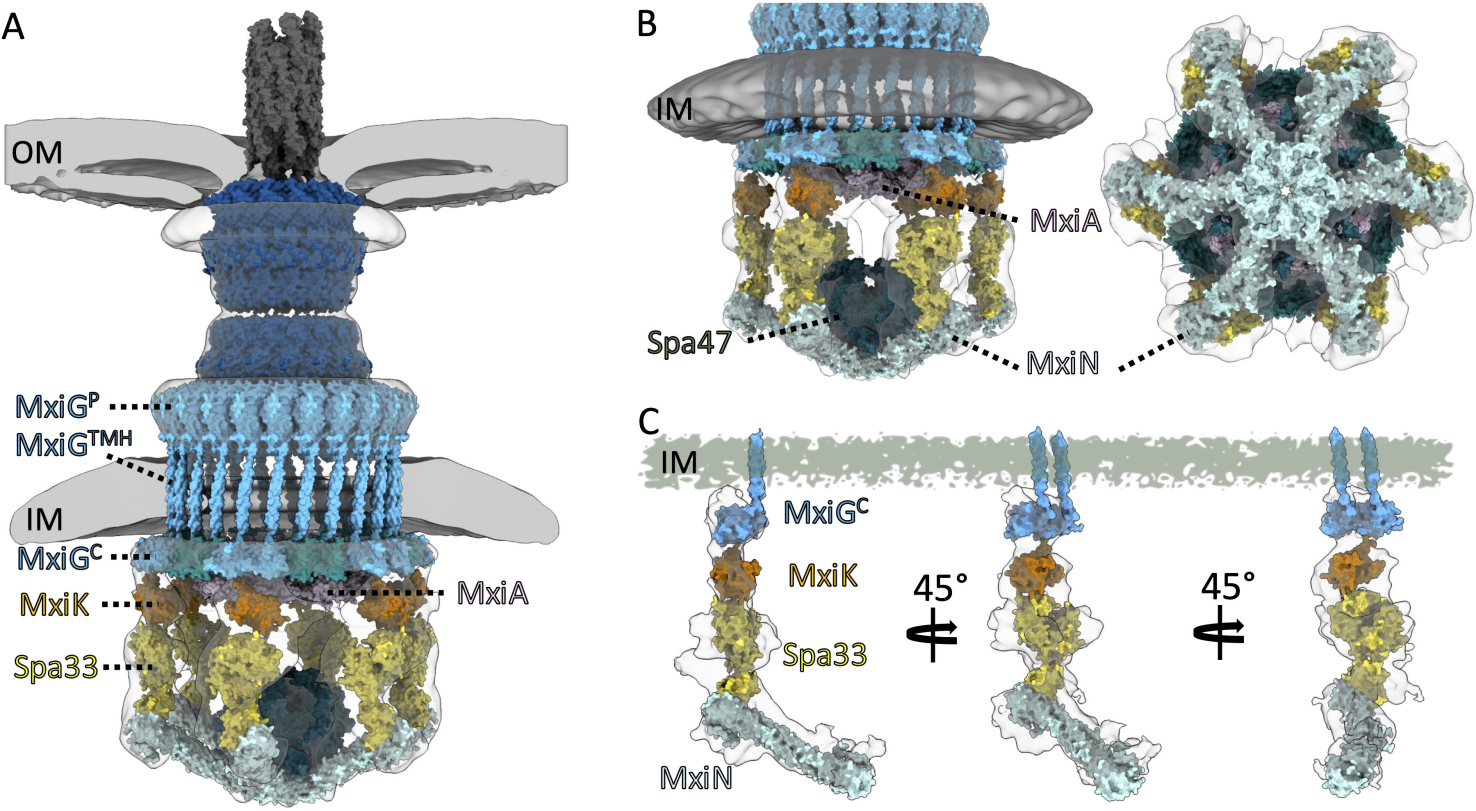
Proposed models are presented for the detailed structure of the *Shigella* injectisome. **(A)** shows the overall architecture of the *Shigella* injectisome from the external needle to the intact cytoplasmic SP. **(B)** shows the Spin detail with the positions of the Spa47 ATPase, MxiA export gate, and the radial spoke protein MxiN indicated. **(C)** shows the individual functional unit connecting the basal body with the Spa47 ATPase. The IR component MxiG is dictated by a pair of MxiG^C^ domains that interact with MxiK that, in turn, interacts with the Spa33 complex. The Spa33 complex continues to be poorly defined which is apparent from the densities of the Spa33 pods that are not accounted for at the molecular level. Spa33 then interacts with the MxiN radial spoke which connects with Spa47 and has a role in guiding the Spa47 ATPase activity. Experimental models of the helical MxiH needle (PDB:6zni), MxiA export gate (PDB:4a5p), and outer membrane complex (PDB: 8axl and 8axn) as well as predicted structures of MxiK monomer, Spa33 hetero-trimer, MxiN homodimer, Spa47 (AF-P0A1C1-F1-v4) hexamer, and a refined model of MxiG were fit into the electro density map (gray and white outlines) from the global averaged structure **(A, B)** and the local refined map (EMD-23440) **(C)**.

The *Shigella* T3SS apparatus or injectisome is this pathogen’s primary virulence factor and is required for invasion of the colonic epithelium. Functional type III secretion requires an external needle (with Ipa-protein tip complex) (Espina et al., 2006; Olive et al., 2007; Picking and Picking, 2016), a basal body that spans both bacterial membranes (having outer and inner membrane rings – OR and IR, respectively) and a cytoplasmic sorting platform (SP) (Hu et al., 2015; Soto et al., 2022). The periplasmic portion of the IR has 24-fold symmetry and is generated from MxiJ (SctJ in the unified nomenclature presented in **Supplementary Table S1**) and a periplasmic domain of MxiG or MxiG^P^ (SctD or SctD^P^) (Hu et al., 2015). MxiG also possesses a transmembrane helix (TMH) and a cytoplasmic domain (MxiG^C^ or SctD^C^) that serves as an anchor for the SP pods via the adaptor protein MxiK (SctK) (Muthuramalingam et al., 2020). MxiG also possesses a flexible linker peptide that connects MxiG^C^ with the TMH. In comparisons between the *in situ* structure of the injectisome and the structure of isolated needle complexes (NCs) from the *Shigella* and *Salmonella* systems, MxiG^C^ (PrgH^C^ in *Salmonella*) is able to move from a position that is in line with the pods of the SP to a uniform position that is shifted inward toward the export gate and channel (Marlovits et al., 2004; Tachiyama et al., 2019). A closer examination of the *in situ* injectisome structure here (**Figure 1**), reveals that MxiG^C^ interacts with MxiK in pairs that are in line with the MxiK/Spa33-containing pods of the SP, however, the intervening pair of MxiG^C^ domains are shifted inward (**Supplementary Figure S3**). This is distinct from what has been observed in *Salmonella* where PrgH^C^ clusters into tetrads where two of the four appear to directly interact with their cognate adaptor protein OrgA (SctK) (Soto et al., 2022). From these observations, we propose that for *Shigella* a single unit connects the basal body to the Spa47 ATPase (**Figure 5**) and this unit consists of two MxiG^C^ associated with a single MxiK, which then associates with a Spa33 (SctQ) complex that remains to be fully characterized. Spa33 then interacts with a MxiN (SctL) dimer that forms the radial spokes that are connected to Spa47 (SctN). The MxiG^C^ pairs that associate with MxiK are in an alignment with MxiK and the bulk of the Spa33 pods, however, the intervening MxiG^C^ pairs are shifted inward toward the interior of the SP (**Figure 1**). This ability for MxiG^C^ to assume multiple positions led us to explore the importance of the MxiG linker within the *Shigella* injectisome.

An important observation from this work is that the MxiG linker can accept a wide range of mutations without loss of injectisome function (**Supplementary Table S2**). The functions tested here included overnight and Congo red (CR) induced secretion of IpaB and IpaD, recruitment of IpaD (needle tip protein) to the *Shigella* surface, translocon formation (measured by contact-mediated hemolysis) and invasion of HeLa cells after brief or extended co-incubation. The mutations introduced into the linker included substitutions, deletions, insertions, and alterations in linker flexibility (**Supplementary Table S2**). Among the deletion mutations, only complete removal of the linker eliminated all injectisome functions. Surprisingly, elimination of linker flexibility with polyproline or a trimer of the peptide EAAAR (a strong α-helix former) failed to eliminate injectisome function. In contrast, an EAAAR dimer (EAAAR) (Ranganathan et al., 2019) and insertion of GFP at the middle of the linker did eliminate all injectisome functions. The MxiG^(EAAAR)2^ mutant did allow for some low-level IpaD surface localization (**Supplementary Figure S7**) and only mildly reduced IpaB secretion (**Supplementary Figure S5**), however, these activities were much lower than what was seen for the MxiG^(EAAAR)3^ mutant. This could be due to length combined with the ultimate orientation of MxiG^C^, however, it is worth noting that four different polyproline mutants which might be expected to give rise to multiple MxiG^C^ orientations never lost more than 50% of the wildtype injectisome function (**Supplementary Table S2**). Meanwhile, insertion of GFP in the middle of the linker eliminated almost all injectisome functions while insertion of bacteriophage T4 lysozyme (T4L) in the middle of the linker or between two full length copies of the linker never reduced contact-hemolysis more than 70% and allowed prominent IpaD presentation of the bacterial surface (**Supplementary Table S2**). This could be a result of MxiG^C^ orientation since the GFP N- and C-termini are located at distal locations, unlike T4L for which the N- and C-termini are located very close together (Barta et al., 2018).

While deletion of the entire linker resulted in complete loss of injectisome function, it should be noted that gradual deletion of residues from the linker resulted in a progressive loss of hemolysis activity until all functions were lost with complete linker elimination (**Figure 2A**). That said, deletion of all but six linker residues only reduced activity by 25% and loss of all but four or three residues resulted in a loss of 40% and 50%, respectively. What is missing from the contact-mediated hemolysis data, however, is what happens when rapid induction of injectisome activity is needed (in this case considered in terms of rapid HeLa cell invasion). We found that loss of all but six residues (MxiG^Δ110-120^) has a significant effect on invasion (p ≤ 0.05), while loss of all but four (MxiG^Δ108-120^) or three (MxiG^Δ111-124^) residues has an even more profound effect on rapid entry into HeLa cells (p ≤ 0.005) (**Figure 3A**). When a subset of mutants was selected for further study, the MxiG^Δ111–124^ mutant (loss of all but three residues) was found to no longer be responsive to rapid Ipa secretion in the presence of CR (**Figure 2C**). These findings indicate that secretion signals propagated from the bacterial surface are not only dependent upon the needle and needle tip complex proteins, but also upon the cytoplasmic linker of the IR protein MxiG. Based on functional testing of the linker mutant library, three MxiG linker mutants were selected for study using cryo-ET, including MxiG^(EAAAR)3^, MxiG^Δ108-124^, and MxiG^Δ111-124^.

The accumulated cryo-ET data show that even in the absence of the entire linker, MxiH (SctF) could still be exported to assemble needles for nearly 25% of injectisomes (**Figure 4**; **Supplementary Figure S8**). The IpaD tip protein, in contrast, was not secreted or found to be present at detectable levels on the bacterial surface (*i.e.* at the tip of the resulting needles), suggesting a loss of substrate switching in the absence of the linker (**Figure 2**; **Supplementary Figure S7**). Interestingly, needle formation occurs even for injectisomes lacking a clearly visible or intact SP based upon subtomogram-averaged structures (**Figure 4E**). This might be interpreted as showing that MxiH export and needle assembly does not require the activity of the Spa47 ATPase, however, when injectisomes were examined for *Shigella* expressing a catalytically inactive Spa47, no needles were present for any of the injectisomes while the SP was fully assembled (see **Supplementary Figure S10**). Meanwhile, a MxiG linker possessing only three residues (MxiG^Δ111-124^) has needles present on nearly 80% of its injectisomes and appears functional, except that it no longer responds to CR signals for induced Ipa protein secretion (**Figure 2C**) and cannot invade HeLa cells in the absence of forced contact with a prolonged incubation (**Figure 3A**). This mutant does possess the SP for both injectisome classes (with and without needles), however, the complex is not clearly resolved in the averaged structures, even though 75% of them assemble the SP.

It is possible that in the absence of the linker, the pods and remainder of the SP form transiently to allow MxiH export, albeit inefficiently, but their instability causes them to immediately disassemble so that no other proteins can be secreted. Therefore, we may be seeing a major shift in the dynamics occurring within the injectisome SP when changes are made to the MxiG linker. Dynamic movements of the SP or its components have been described previously (Diepold et al., 2015; Diepold et al., 2017; Wimmi et al., 2024) and mutations within the SP pod component can lead to a loss of SP formation in *Salmonella* without a loss of T3SS secretion (Lara-Tejero et al., 2019). Furthermore, dynamic movements have even been seen within the T3SS injectisome basal body (Wimmi et al., 2021). In the same respect, the formation of a recognizable and stable SP does not guarantee that type III secretion will occur (**Figure 4**, **Supplementary Figures S9**, **S10**) and the absence of a stable and visible SP in cryoelectron tomograms does not mean that T3SS early substrates (*i.e.* MxiH) will not be secreted (**Figure 4**; **Supplementary Figure S9**).

Based on the findings presented here, we propose that the MxiG linker must be long enough and flexible enough to allow rapid activation of the T3SS in response to secretion signals received at the needle tip. Altering the linker affects secretory function by making it more difficult for MxiG to bind the MxiK adaptor in an orientation amenable to the correct assembly of other SP components. MxiK must not only bind MxiG, but also Spa33, in a complex that is roughly perpendicular to the inner membrane (see **Figure 5**). The MxiG/MxiK interaction can be thought of in the context of the equilibrium:


G1+G2+K ⇌G1+G2K⇌G1G2K⇌G1G2K∗

Where G1 and G2 are MxiG cytoplasmic domains, K is MxiK, and * indicates the conformation required for subsequent SP assembly and stability. Mutations to the linker that decrease its length or make it more rigid shift the equilibrium away from the starred conformation, either by destabilizing it or making the MxiG/MxiK complex less likely to adopt it. When the linker is fully deleted, the SP competent conformation is very rarely sampled, such that the SP is assembled so transiently that it can only secrete some MxiH before disassociating. Mutants that fail to rapidly respond to secretion signals but retain hemolytic and overnight secretory function likely cannot convert to the competent conformation rapidly, but once they do the SP can form and remain assembled long enough to form translocons and secrete effectors.

What is not investigated here, but which is alluded to, is the role of the export gate in secretion induction in *Shigella*. The position of the MxiA cytoplasmic domain appears to correlate with secretion activity, however, a focus on this injectisome component will require additional work. The injectisome structures formed for the MxiG^Δ108–124^ mutant in this study suggest that the MxiA export gate is shifted upward and the basal body has the central export channel blocked when the injectisome is fully inactive (**Figure 4F**; **Supplementary Figure S9F**). A full understanding of type III secretion will require teasing out the shared and, in turn, distinct features of injectisomes from related and perhaps less closely related systems. The T3SS of *Shigella*, *Salmonella* and *Yersinia* have been studied extensively, but a full understanding of injectisome mechanics may require expanding our explorations to include more distantly related systems, perhaps even the T3SS from plant pathogens/symbionts so that we can fully appreciate the importance of architectural features and dynamic motions within a wide range of injectisomes.

## Materials and methods

### *Shigella* strains

*S. flexneri* serotype 5a (M90T) and a *mxiG* null mutant strain were from John Rohde (Dalhousie University, Halifax, NC, Canada). Minicells of *S. flexneri mxiG* null strains expressing wildtype or mutant *mxiG* were generated by introducing plasmid pBS58, which constitutively expresses *Escherichia coli* cell division genes *ftsQ*, *ftsA* and *ftsZ* from a low-copy, spectinomycin-resistant plasmid (Hu et al., 2015). Spectinomycin (100 µg/ml) was added for selection of pBS58, and 50 µg/ml kanamycin to select for the parent *mxiG* null mutant and 100 µg/ml ampicillin to select for the plasmid expressing mutant *mxiG* in pWPsf4 plasmid (Picking et al., 2005). Bacterial cultures were grown at 37°C to late log phase (A_600_ 0.8). To enrich for minicells, the culture was centrifuged at 1000×g for 5 min to remove the large cells, and the supernatant fraction was further centrifuged at 20,000×g for 10 min to collect the minicells. The *Shigella* strain expressing an inactive mutant of the Spa47 ATPase was provide by Nicholas Dickenson (Utah State University, Logan, UT) (Burgess et al., 2016).

### Cloning of the MxiG mutant library

Primers for nucleotides that encoded strings of Ala were designed to replace specific regions within MxiG using inverse PCR. The wildtype *mxiG* in pWPsf4 was used as a template for inverse PCR products containing homologous nucleotides at 5’ and 3’ ends. The agarose gel electrophoresis and QIAquick gel extraction kit (QIAGEN) was used to purify the PCR products, which were then digested with DpnI (New England Biolabs). The reaction mixtures were purified by QIAquick PCR purification kit (QIAGEN). The final product was treated with 5× In-Fusion HD Enzyme Premix (Takara Bio) to ligate the 5’ and 3’ ends. The reaction mixtures were transformed into the competent *E. coli* cells for DNA sequence confirmation and the resulting pWPsf4-mxiG plasmids were used to transform a *Shigella mxiG* null strain. The primers used in this study are described in **Supplementary Table S5**. The relative expression levels and stability of the key MxiG mutants produced in *Shigella* was tested by immunoblot analysis (**Supplementary Figure S11**).

### Overnight steady state and Congo red induced secretion

The secretion activity of T3SS was tested by detecting IpaB and IpaD levels in the overnight bacterial cultures (Espina et al., 2006; Lu et al., 2023). Colonies of *S. flexneri* and its various mutants were taken from trypticase soy agar (TSA) plates containing CR, ampicillin (Amp), and kanamycin (Kan) and inoculated into trypticase soy broth (TSB) with Amp and Kan. The bacterial cultures were grown at 37 °C overnight. The supernatant fractions were collected by centrifugation and the proteins in the supernatant fraction were precipitated with 10% trichloroacetic acid (TCA) and then centrifuged at 10,000 rpm for 15 min at 4 °C to collect protein precipitants. The precipitants were washed in 5% TCA and then iced cold acetone. After the evaporating acetone, the precipitated proteins were resuspended in 400 µL 10 mM phosphate (pH 7.2) with 150 mM NaCl (PBS) to use for the immunoblot analysis. The proteins were separated for the immunoblot on 10% polyacrylamide gels that were blotted on nitrocellulose membranes. Mouse serum containing anti-IpaB and anti-IpaD IgG (Lu et al., 2023) was used as the primary antibody with rabbit anti-mouse IgG with an infrared tag (Li-Cor, Lincoln, NE) used to detect the IpaB and IpaD. Densitometry was used to quantify the relative amounts of IpaB secreted by each strain.

For CR induced secretion, bacterial were grown to mid log phase (A_600_ 0.6) and collected by centrifugation. The bacteria were resuspended in one tenth volume PBS and CR added to a final concentration of 0.025%. After 20 min at 37 °C, the bacteria were pelleted by centrifugation and the supernatants examined for the presence of IpaB and IpaD as for overnight secretion analysis.

### Contact-mediated hemolysis

*Shigella* strains were streaked on CR plates with appropriate antibiotics for measuring contact-mediated hemolysis (Picking et al., 2005). Several red colonies from the plates were inoculated on 10 mL of TSB with the appropriate antibiotics and grown to mid-log phase (OD600 ~ 0.6) and OD values recorded. The bacterial cultures were centrifuged at 3756×g for 15 min at 30 °C, and then the bacterial pellets were resuspended by PBS to an OD of 20. Red blood cells (50 µL) at 4×10 (Espina et al., 2006) cells/mL was mixed with 50 µL of the *Shigella* samples in a round bottom micro-plate. The microplate was then centrifuged at 2,876×g for 15 min at 30°C. The micro-plate was incubated at 37°C for 60 min. Ice-cold PBS (100 µL) was used to resuspend the mixed bacteria and red blood cell pellets, and centrifuged at 2,876×g for 15 min at 10°C. The supernatants (100 µL) were transferred to a new round bottom micro-plate, and then the relative hemoglobin levels determined by absorbance at 545nm (A_545_).

### *Shigella* invasion of HeLa cells

*Shigella* MxiG linker variants were grown with shaking in 10 mL tryptic soy broth (TSB) at 37°C until they reached an OD600∼0.6 for use in HeLa cell invasion assays (Picking et al., 2005). Cultures were then centrifuged at 2000×g for 10 min, the media supernatant removed, and cell pellets resuspended in 5 mL of Eagle’s minimum essential medium (EMEM) containing 0.4% glucose. The A_600_ of each resuspension was measured and 10^8^ cells of each variant were added to each of three wells of a 24-well plate containing confluent (~2×10^6^) HeLa cells. EMEM with glucose was then added to each well to reach a final volume of 0.5 mL. At this point, the 24 well plate was either a) centrifuged at 1000×g for 10 min and then incubated at 37°C for 1 h, or b) not centrifuged and incubated at 37°C for 30 min. In each case, incubation was performed in a 5% CO_2_ environment. Following incubation each well was washed twice with 0.5 mL EMEM containing 100 μg/mL gentamycin. Each well was then filled with 0.5 mL of EMEM with gentamycin and incubated for 1 h at 37°C with 5% CO_2_. The media was then aspirated from each well and the HeLa cells were lysed by the addition of 0.5 mL PBS containing 1% Triton X-100 followed by mechanical scraping of the well bottom with a pipet tip for 30 sec. Volumes of 100, 10, and 1 μL were then plated on TSB plates. CFUs were counted following overnight incubation of plates at 37°C.

### Cryo-ET sample preparation

For cryo-ET sample preparation, a plasmid which possesses *ftsQ*, *ftsA*, and *ftsZ* from *E. coli* was introduced into each *Shigella* strain using the electroporation. Each *Shigella* strain was streak on TSA plates with appropriate antibiotics and incubated at 37°C for overnight. A single colony was inoculated in 10 mL of TSB with the antibiotics, and then grown overnight at 37°C. Two mL of the overnight culture was inoculated in 200 mL of TSB with the appropriate antibiotics for grow to late log phase. The bacterial culture was centrifuged with 2,000×g 4°C for 10 min twice. The bacterial supernatant was then centrifuged at 20,000×g at 4°C for 10 min. Minicell pellets were then resuspended in PBS and centrifuged at 1500×g for 5min to remove large debris. The sample was then centrifuged at 15,000×g for 5 min and the minicell pellets were resuspended in 20 µL PBS. These *Shigella* minicell samples were mixed with 10 nm BSA coated gold tracers (Aurion), and then 5 µL of each specimen was deposited on a glow-discharged copper cryo-EM grid (Quantifoil R2/1 Cu 200 mesh). To prepare frozen-hydrate *Shigella* minicell specimens, the grids were blotted with filter paper and immediately plunged into liquid ethane using a home-made gravity-driven plunger.

### Cryo-ET data collection and processing

A Glacios electron microscope (Thermo Scientific) equipped with a field emission gun and a Direct Detection Camera (K2 summit, Gatan) was used to image the frozen-hydrate specimens at ~ -170°C. The microscope was operated at 200 kV using SerialEM software (Mastronarde, 2005). The minicell images were recorded at the magnification with a physical pixel size of 2.40Å. The stage angles ranged from -48° to +48° in increments of 3°, and the tilt series images were recorded using the dose-symmetric scheme in FastTomo script (Xu and Xu, 2021). A total electron dose of 70 e^-^/Å (Ranganathan et al., 2019) was distributed among 33 stacks in the tilt series. The dose-fractionated mode in SerialEM was used to record 10 frames in each image. Motioncor2 (Zheng et al., 2017) was used to correct image drifting caused by the electron beam during the image recording. Then, IMOD software was used to create image stacks of tilt series and align all images in each image stack by tracking the gold tracers (Kremer et al., 1996; Mastronarde and Held, 2017). Defocus for all images in each stack was estimated using Gctf (Xiong et al., 2009; Mastronarde and Held, 2017), and contrast transfer function (CTF) was corrected using ctfphaseflip function in IMOD (Xiong et al., 2009). After alignment of images in tilt series, binvol command in IMOD was used to generate 8× binned aligned stacks. Then, 8× binned tomograms with Simultaneous Iterative Reconstruction Technique (SIRT) was reconstructed by Tomo3D software (Agulleiro and Fernandez, 2015). Injectisomes in tomograms were manually selected, and then selected particles were used for the subtomogram averaging. Based on aligned positions in 8× binned tomograms with Weighted Back Projection (WBP), subtomograms were extracted, and 2× and 4 ×binned subtomograms were generated using binvol command in IMOD (Xiong et al., 2009). After refinement of the structures in the 4× binned subtomograms, 3-D classification was used to classify the two classes of injectisomes (those with and those without a visible MxiH needle) to determine the *in situ* structures of the injectisomes (**Figure 4**). A second 3-D classification was also performed to classify injectisome with and without densities corresponding to the sorting platform components (**Figure 4**, Top; **Supplementary Figure S9**). All *in situ* structures of the injectisome were determined by i3 software package (Winkler, 2007; Winkler et al., 2009; Morado et al., 2016). To measure the length of needles, tomograms with SIRT from wildtype and each key mutant were imported in Dragonfry software. Then, 10 injectisomes from each strain were randomly selected to measure distance between the outer membrane and tip of the needle.

### Flow-cytometry of *Shigella* MxiG mutants

*S. flexneri* mxiG linker mutants were streaked on CR-containing TSA plates containing 50 µg/ml Kan to select for the parent *mxiG* null strain and 100 µg/ml Amp to select for the pWPsf4 plasmid expressing the *mxiG* linker mutants. A single colony was inoculated in TSB media with the appropriate antibiotics and grown at 37°C to an A_600_ of 0.8. Cells were gently rinsed with and then chemically fixed in 4% formaldehyde prepared in PBS buffer for 15 min in the dark at room temperature. Cells were rinsed and then resuspended in FACS buffer (2% fetal bovine serum in PBS). Cells were labeled with rabbit antibodies against IpaD (1:1000 dilution) for 1 hour. The cells were then washed with FACS buffer and spun down. The pellet was resuspended in FACS buffer with Alexa647 goat anti-rabbit secondary antibody (1:200 dilution) and incubated for 30 min on ice. The cells were then washed with FACS buffer and pelleted by centrifugation. Lastly, the cells were resuspended in FACS buffer and acquisitions were performed on flow cytometer (BD FACSAria Fusion) collecting 100,000 instances/run.

## Data Availability

The datasets presented in this study can be found in online repositories. The names of the repository/repositories and accession number(s) can be found below: https://www.ebi.ac.uk/pdbe/emdb/, EMD-70158 https://www.ebi.ac.uk/pdbe/emdb/, EMD-70160 https://www.ebi.ac.uk/pdbe/emdb/, EMD-70161 https://www.ebi.ac.uk/pdbe/emdb/, EMD-70162 https://www.ebi.ac.uk/pdbe/emdb/, EMD-70165 https://www.ebi.ac.uk/pdbe/emdb/, EMD-70166.
