# Supplementary material for: A flexible peptide linking the periplasmic and cytoplasmic domains of MxiG controls type III secretion signaling and stable sorting platform assembly in Shigella
Source: Front Cell Infect Microbiol. 2025 Aug 4;15:1611779. doi: 10.3389/fcimb.2025.1611779 (PMC12358396; doi:10.3389/fcimb.2025.1611779)
Supplement: Supplementary file 1 [file Table1.docx]

**Supplemental Materials for. . .**

**A flexible peptide linking the periplasmic and cytoplasmic domains of MxiG controls the efficiency of type III secretion in *Shigella***

Shoichi Tachiyama^1,2^, Meena Muthuramalingam^3^, Sean K, Whittier^4,5^, Yunjie Chang^1,2^, Jian Yue^1,2^, Waleed Younis^4,5,6^, Wendy L. Picking^4,5^, Jun Liu^1,2^* and William D. Picking^4,5^*

^1^Department of Microbial Pathogenesis, Yale University West Haven, CT 06516, ^2^Microbial Sciences Institute, Yale University, West Haven, CT 06516, ^3^Department of Pharmaceutical Chemistry, University of Kansas, Lawrence, KS 66045, ^4^Department of Veterinary Pathobiology, ^5^Department Christopher S. Bond Life Sciences Center, University of Missouri, Columbia, MO 65211, and ^6^Department of Microbiology, Faculty of Veterinary Medicine, South Valley University, Qena, Egypt 83523

*Corresponding authors: [pickingw@missouri.edu](mailto:pickingw@missouri.edu) and jliu@yale.edu

**Supplemental Figure S1.** The **Left** side shows the steps of *Shigella* invasion of an epithelial cells beginning with invasion (lower right), vacuolar escape (top) and intracellular motility which precedes intercellular spread. The **Right** side is a cartoon of the *Shigella* T3SS injectisome showing the sorting platform (SP) components from inner membrane ring (MxiG/SctD) to the adaptor protein (MxiK/SctK) to the pods (Spa33/SctQ), radial spokes (MxiN/SctL) and the central ATPase (Spa47/SctN). Inside the SP “cage” is the export gate (MxiA/SctV) whose cytoplasmic domain is visible in the cryo-ET images presented here.

**Supplemental Table S1. Unified Nomenclature for the Type III Secretion System Needle, Basal Body and Sorting Platform Components**

Injectisome

Component *Shigella* *Salmonella* *Yersinia*  Unified Role /Location

**Needle** MxiH PrgI YscF SctF Needle monomer

**Basal Body Rings** MxiD InvG YscC SctC Secretin

MxiJ PrgK YscJ SctJ Inner IM ring

MxiG PrgH YscD SctD Outer IM ring

MxiI PrgJ YscI SctI Inner rod

**Basal Body and** Spa24/SpaP SpaP YscR SctR Export apparatus

**Accessory Proteins** Spa9/SpaQ SpaQ YscS SctS “ “

Spa29/SpaR SpaR YscT SctT “ “

Spa40/SpaS SpaS YscU SctU “ “

MxiA InvA YscV SctV Export gate

MxiC InvE YopN SctW Gatekeeper

Spa32 InvJ YscP SctP Needle length control

**Sorting Platform** MxiK OrgA YscK SctK Adaptor

Spa33 SpaO YscQ SctQ Pod protein

MxiN OrgB YscL SctL Radial spoke

Spa47 InvC YscN SctN ATPase

Spa13 InvI YscO SctO Stalk protein

The unifying nomenclature is based upon the names used in the *Yersinia* (Ysc) system for the actual injectisome (S. Wagner and A. Diebpld (2020) A unified nomenclature for injectisome-type type III secretion systems. Curr Top Microbiol Immunol. 2020;427:1-10. doi: 10.1007/82_2020_210. PMID: 32415388).


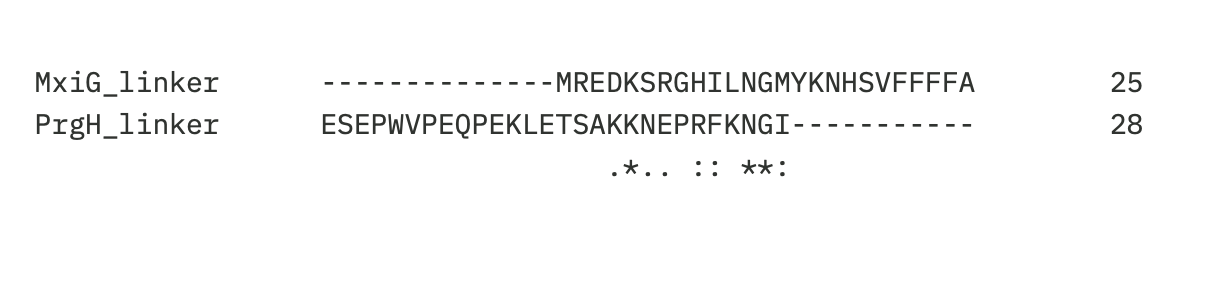


**Supplemental Figure S2.** An alignment of the linker regions of *Shigella* MxiG and *Salmonella* PrgH are shown.


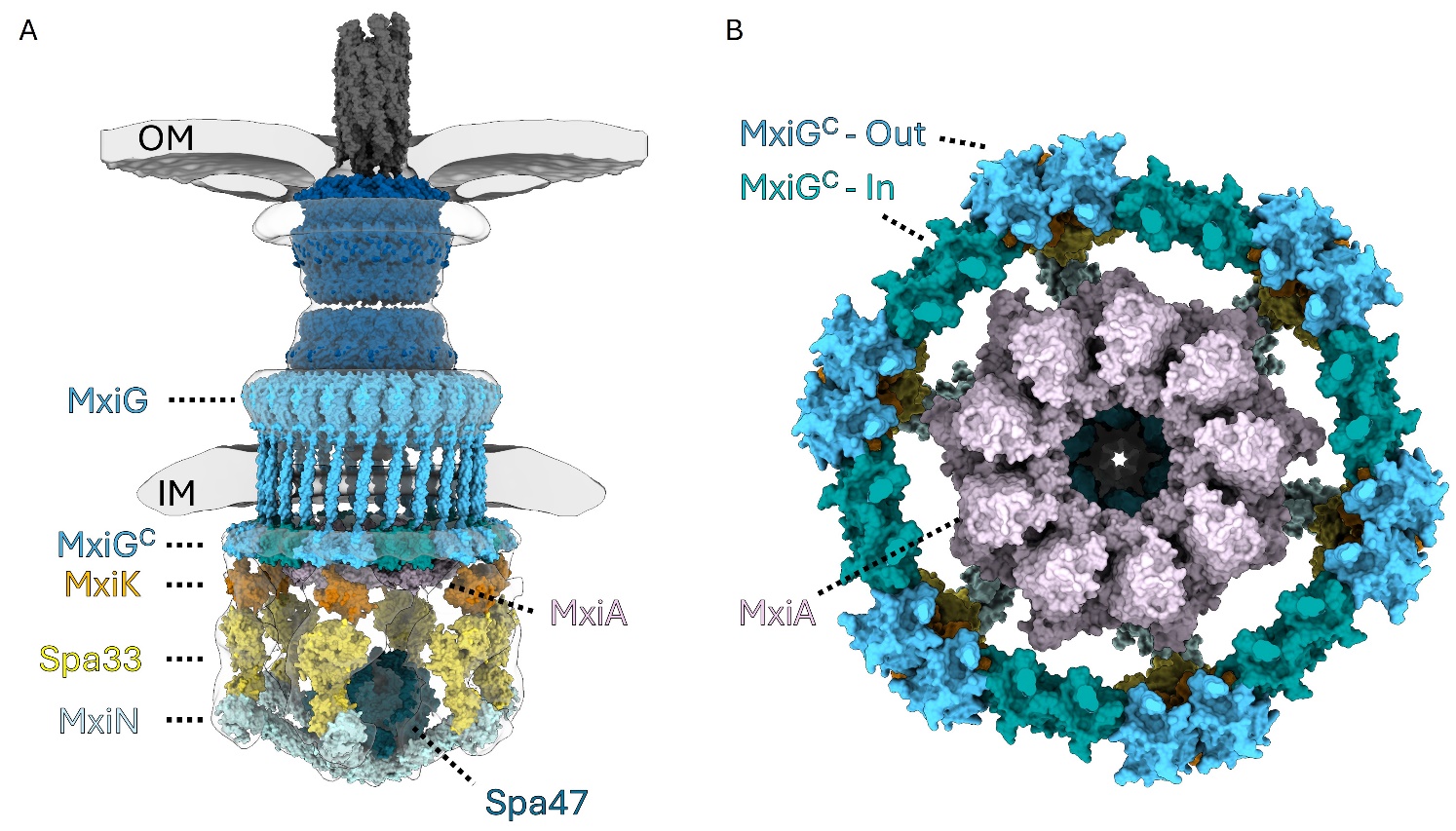


**Supplemental Figure S3.** An updated 3D rendering of the pseudo-atomic structures of the injectisome components with the SP components indicated. The helical MxiH needle (PDB: 6zni), outer membrane complex (PDB: 8xal and 8axn), and MxiA export gate (4a5p) were docked into the density map. Predicted structures of MxiK, Spa33, MxiN, and Spa47 (AF-P0A1C1-F1-v4) and refined model of MxiG were also docked into the map. On the left is the overall injectisome structure, including periplasmic and extracellular structures. The right depicts the positions of the cytoplasmic components of the export gate (MxiA) and the IR. To visualize models clearly, the electron density map was removed from this model. The positions shown for the 24 MxiG^C^ domains is based upon the positioning shown in **Figure 1**. The rendering on the right is based upon a top-down view so that the radial spokes (MxiN) are seen in the background.

**Supplemental Table S2.**

% Contact IpaD surface Relative

Classification Mutation* Mutant name** Hemolysis^1^ staining (% of cells)^2^ Invasion^3^

NA *mxiG* null MxiG^-^ 0 0 0/0

NA *mxiG*^_^/*mxiG+* MxiG^+^ 100 96.5 100/100

Substitutions G115P, G120P 100 97.7 -

Pro15 Linker 60 90.0 -

Pro16 Linker 63 91.3 -

Pro17 Linker 54 84.7 -

Pro18 Linker 59 82.0 -

(EAAAR)_2­_ α2 (Alpha-2) 4 7.9 -

(EAAAR)_3_ α3 (Alpha-3) 73 98.3 74/38

PrgH Linker 100 96.7 -

Deletions ΔG115/G120 100 88.1 -

ΔG115 to G120 100 96.4 -

ΔE110 to G120 Δ110-120 75 93.9 54/46

ΔM108 to G120 Δ108-120 60 98.7 48/66

ΔD111 to N124 Δ111-124 50 98.6 6/65

ΔM108 to N124 Δ108-124 (ΔLinker) 0 0 0/0

Insertions N124-(Gly)_5_-H125 90 97.0 -

N124-(Gly)_10_-H125 70 94.3 -

(Linker)_2_ 2xLinker 80 97.2 76/40

G115-(T4L)-H116 40 98.8 -

Linker-T4L-Linker 30 98.5 -

G115-(GFP)-H116 0 4.3 -

*The reasoning for the generation of these mutants was as follows:

Substitution mutants: Proline residues were used to replace Glycine residues to introduce increased rigidity at those point displaying the greatest flexibility. The poly-proline sequences were used to replace the entire linker to give linkers with highly rigid structures, however, flexibility still possible at each end of the poly-proline sequence. The sequence EAAAR represents a sequence with a strong propensity for forming α-helices. Two and three tandem EAAAR sequences were used to replace the linker to provide a linker that was rigid with somewhat less flexibility at each end compared to the poly-proline sequences. Alpha-2 and Alpha-3 were generated to give two different lengths of linker replacement that would likely hold MxiG^C^ in slightly different orientations. PrgH substitution was generated to determine how the replacement of the linker with that from its *Salmonella* homologue PrgH affected MxiG activity.

Deletion mutants: Deletion mutants were introduced to reduce the distance between the MxiG TM helix and the MxiG^C^ FHA domain. The size of the different deletions (from top to bottom) was 2, 6, 11, 13, 14 and 17 residues with the last deletion mutant lacking the linker entirely.

Insertion mutants: The insertion mutants were designed to determine the effect of large changes in the linker makeup on the ability for the mutant to complement a *Shigella* *mxiG* null strain. Small insertions introducing increased flexibility included poly-glycine segments and two tandem linker sequences were introduced to provide a linker having an exaggerated length. To introduced complex new structures within the linker, inactive bacteriophage T4 lysozyme (T4L) was introduced in the middle of the wild-type linker (after residue 115) or between the two tandem linkers. Green fluorescent protein (GFP) was also introduced at the middle of the wild-type linker to provide even greater complexity.

**Designated names were given to mutants that were selected for more in-depth analysis here. These names are used throughout the text of the manuscript.

^1^Conact-mediated hemolysis, ^2^surface presentation of IpaD (as determined by flow cytometry) and ^3^invasion of cultured HeLa cells was measured following a 30 min incubation without centrifugation (first value) or following a 60 min incubation with centrifugation (second value) to determine whether each mutant could complement T3SS functions when expressed in the *mxiG* null *Shigella* strain used here.


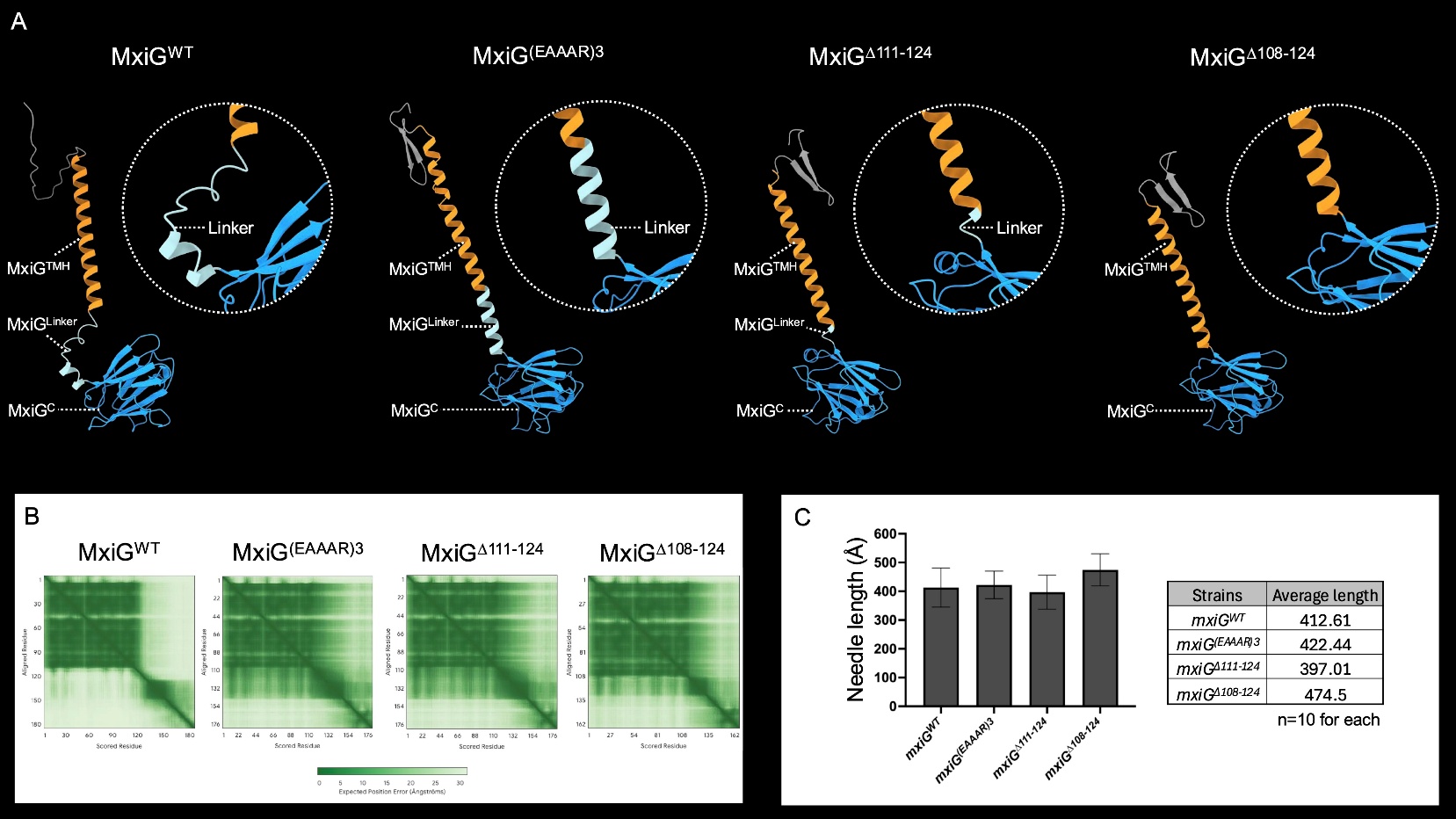


**Supplemental Figure S4.** AlphaFold prediction for the transmembrane helix and globular MxiG^C^ domain of wildtype MxiG, MxiG^(EAAAR)^, MxiG^Δ111-124^, and MxiG^Δ108-124^ (**panel A**). It is worth noting that the predicted aligned error between MxiG^C^ (top left block, **panel B)** relative to the transmembrane region (lower right block, **panel B**) decreases when the (EAAAR)^3^ sequence is used to replace the linker or when most or all of the linker is deleted (**panel B**). **Panel C** shows the relative lengths of the needles from *Shigella* mini-cells expressing wildtype MxiG, MxiG^(EAAAR)3^, MxiG^Δ111-124^, and MxiG^Δ108-124^. In this case, 10 needles were randomly selected for determining length (expressed in Å).

AlphaFold was used to generate the structures in **Panel A**:

(Abramson J, et al. Accurate structure prediction of biomolecular interactions with AlphaFold3. Nature. 2024;630:493-500. doi: <https://doi.org/10.1038/s41586-024-07487-w>

and

Jumper J, et al. Highly accurate protein structure prediction with AlphaFold. Nature. 2021;596:583–589. doi: 10.1038/s41586-021-03819-2

and

Mirdita M et al. ColabFold: making protein folding accessible to all. Nature Methods. 2022;19:679-682. doi: <https://doi.org/10.1038/s41592-022-01488-1>)

Dragonfly was used to determine the needle lengths in **Panel C**:

(Dragonfly 2024.1 [Computer software]. Comet Technologies Canada Inc., Montreal, Canada; software available at [https://dragonfly.comet.tech/](https://nam02.safelinks.protection.outlook.com/?url=https%3A%2F%2Fdragonfly.comet.tech%2F&data=05%7C02%7Cpickingw%40missouri.edu%7Cd44b33bf138c401ef3e008dd9d4ac728%7Ce3fefdbef7e9401ba51a355e01b05a89%7C0%7C0%7C638839666968949200%7CUnknown%7CTWFpbGZsb3d8eyJFbXB0eU1hcGkiOnRydWUsIlYiOiIwLjAuMDAwMCIsIlAiOiJXaW4zMiIsIkFOIjoiTWFpbCIsIldUIjoyfQ%3D%3D%7C0%7C%7C%7C&sdata=1I2BHwS4wrp8zoax2VZ8Lsf3ZBXZ%2B7WOEUGP%2B7Ox%2FPo%3D&reserved=0))


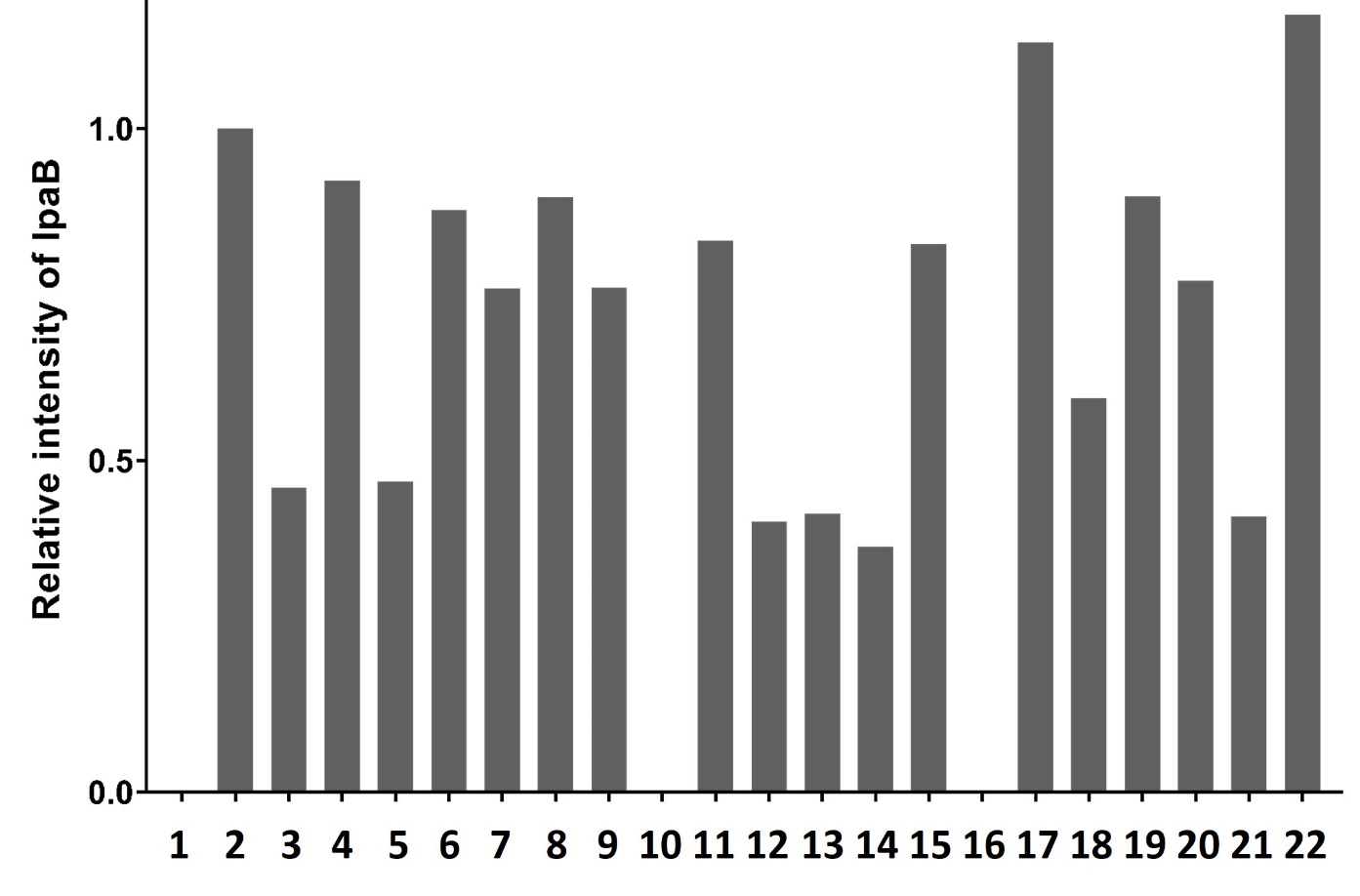


**Supplemental Figure S5.** Densitometric values for IpaB from immunoblots is shown for the mutants described in **Supplementary Table S1**. Only the *mxiG* null strain, the strain expressing *mxiG Δ108-124* (*ΔLinker*) and MxiG with GFP inserted are completely negative for IpaB staining. The mutants in order from 1 to 22 are as follows: 1. *mxiG^-^*, 2. *mxiG^-^/mxiG^+^*, 3. G115P/G120P, 4. N124-(Gly)_5_-H125, 5. ΔG115/G120, 6. ΔG115-G120, 7. N124-(Gly)_10_-H125, 8. ΔE110-G120, 9. PrgH Linker, 10. ΔM108-N124, 11. (Linker)_2_, 12. ΔM108-G120, 13. ΔD111-N124, 14. Linker-T4L-Linker, 15. G115(T4L)H116, 16. G115-(GFP)-H116, 17. Pro15 Linker, 18. Pro16 Linker, 19. Pro17 Linker, 20. Pro18 Linker, 21. (EAAAR)_2­_, 22. (EAAAR)_3_. This figure is a representative profile for secretion by these mutants.

**
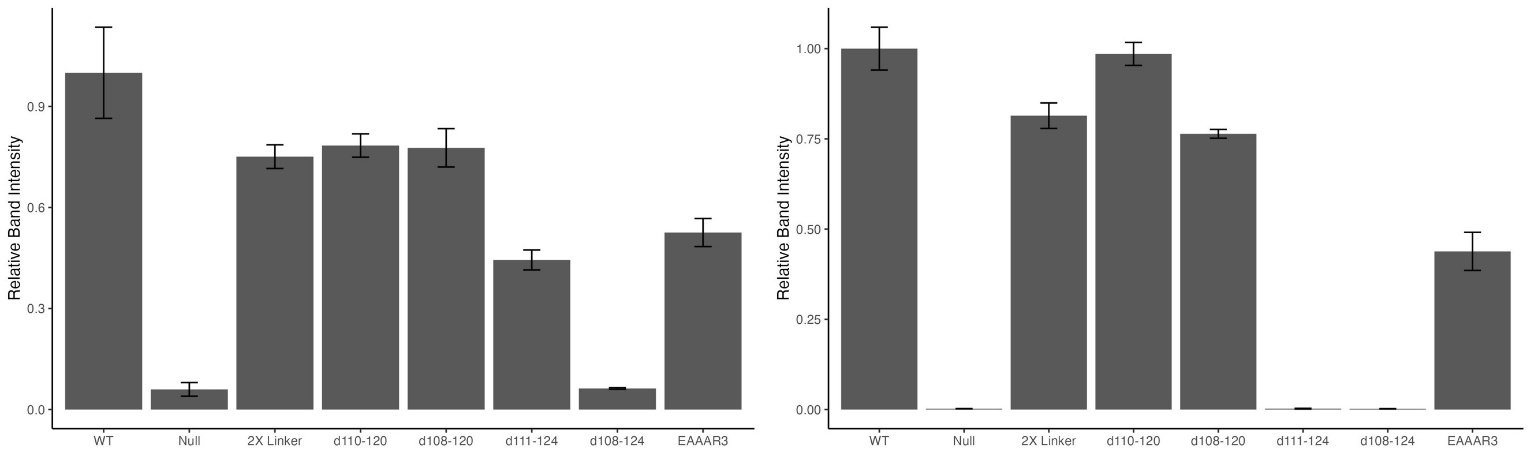
**

**Supplemental Figure S6.** Densitometric values specifically for IpaB from the immunoblots shown in **Figure 2**. Band intensities for selected MxiG linker variants. Overnight secretion (left) and CR-induced secretion (right), along with immunoblotting were performed as described in Methods. Band intensities were quantified using ImageJ software. Reported intensities are the areas of luminosity peaks obtained from equally sized slices through each gel band. All values are normalized to the average intensity of IpaB secreted by *Shigella* expressing wildtype MxiG.


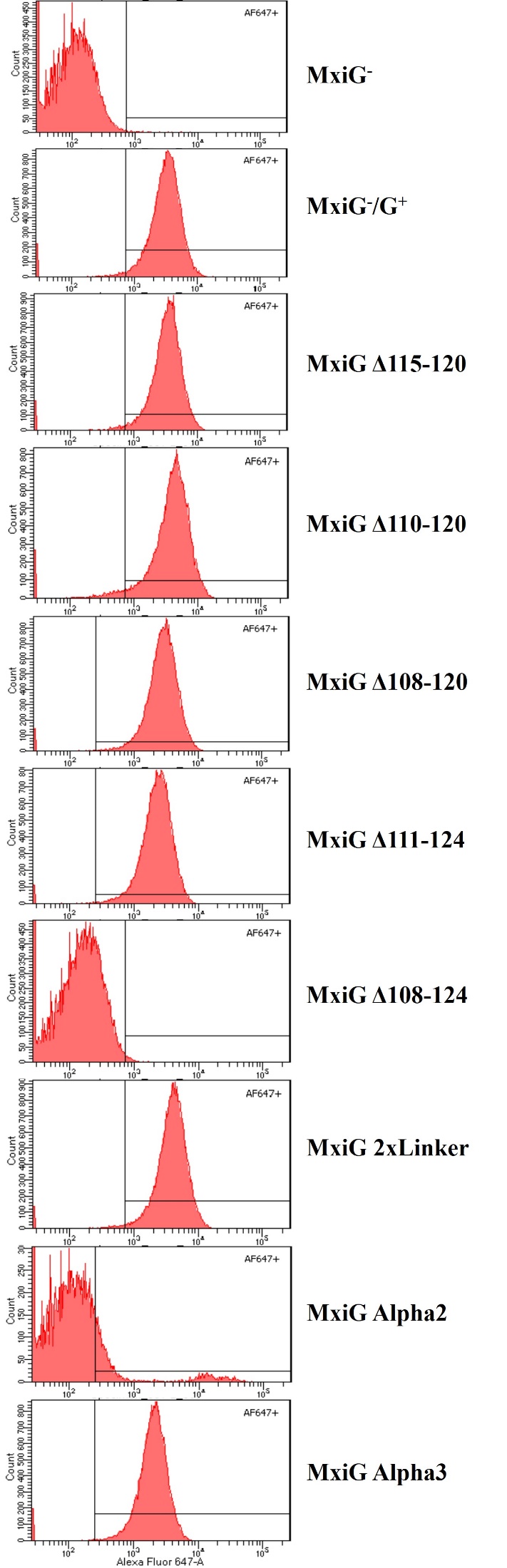


**Supplemental Figure S7.** Flow cytometry of whole *Shigella* is shown after staining for IpaD on the bacterial surface. The percentage of cells staining positive for IpaD on the surface is summarized in **Table S2**. (Alpha2 = (EAAAR)^2^ and Alpha3 = (EAAAR)^3^)


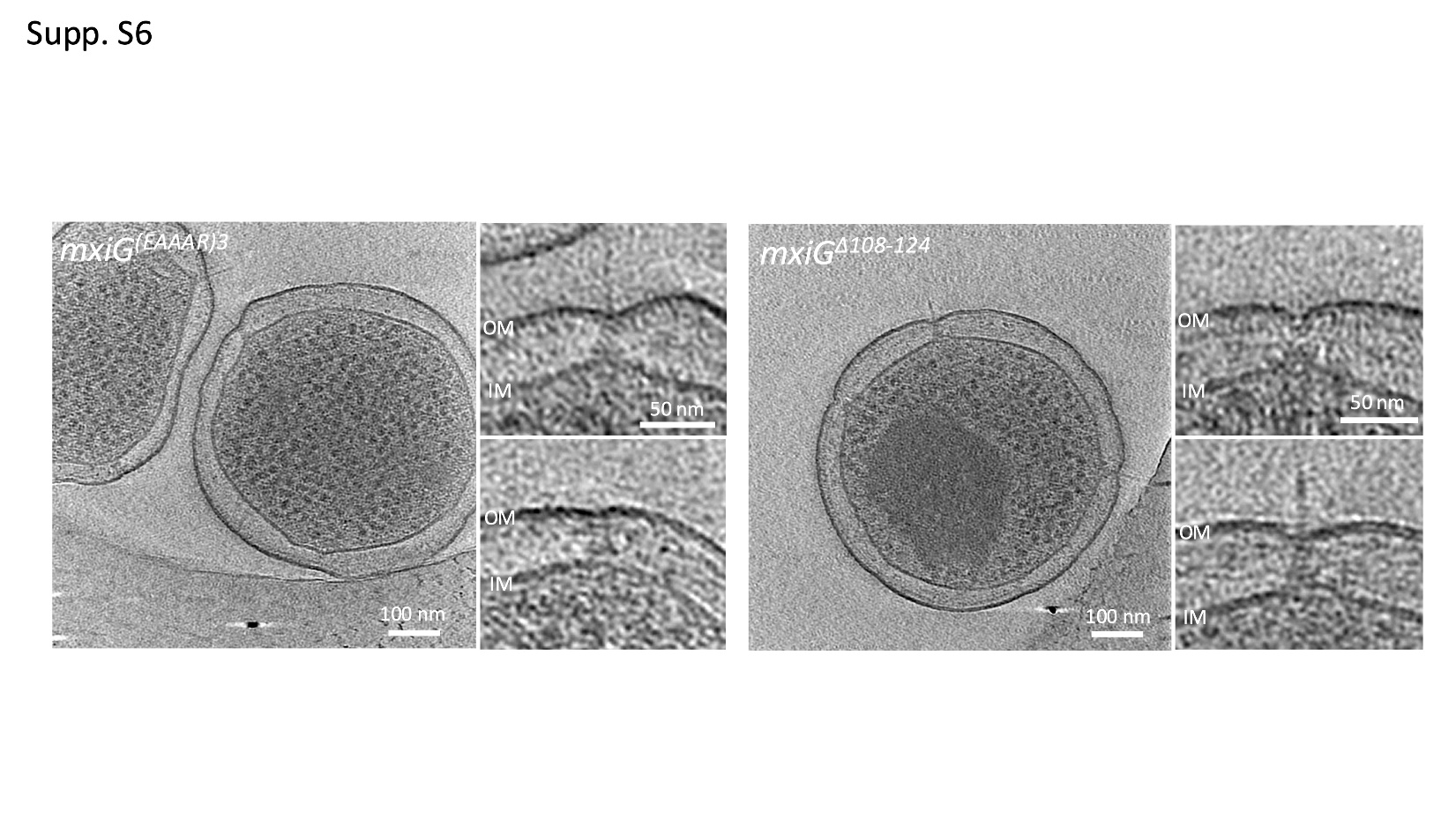


**Supplemental Figure S8.** Based on cryo-ET analyses, the selected *Shigella* mutants could be divided into two groups with one population possessing visible needles and one lacking visible needles. Examples are shown above with the MxiG^(EAAAR)3^ mutant at the **Left** with two examples shown to its the **right**. The top is an example of an injectisome with a needle and the bottom is an injectisome lacking a needle. The MxiG^Δ108-124^ is on the **Right** with two examples shown to its right. The top is an example of an injectisome lacking a needle and the bottom is an example of an injectisome with a needle.


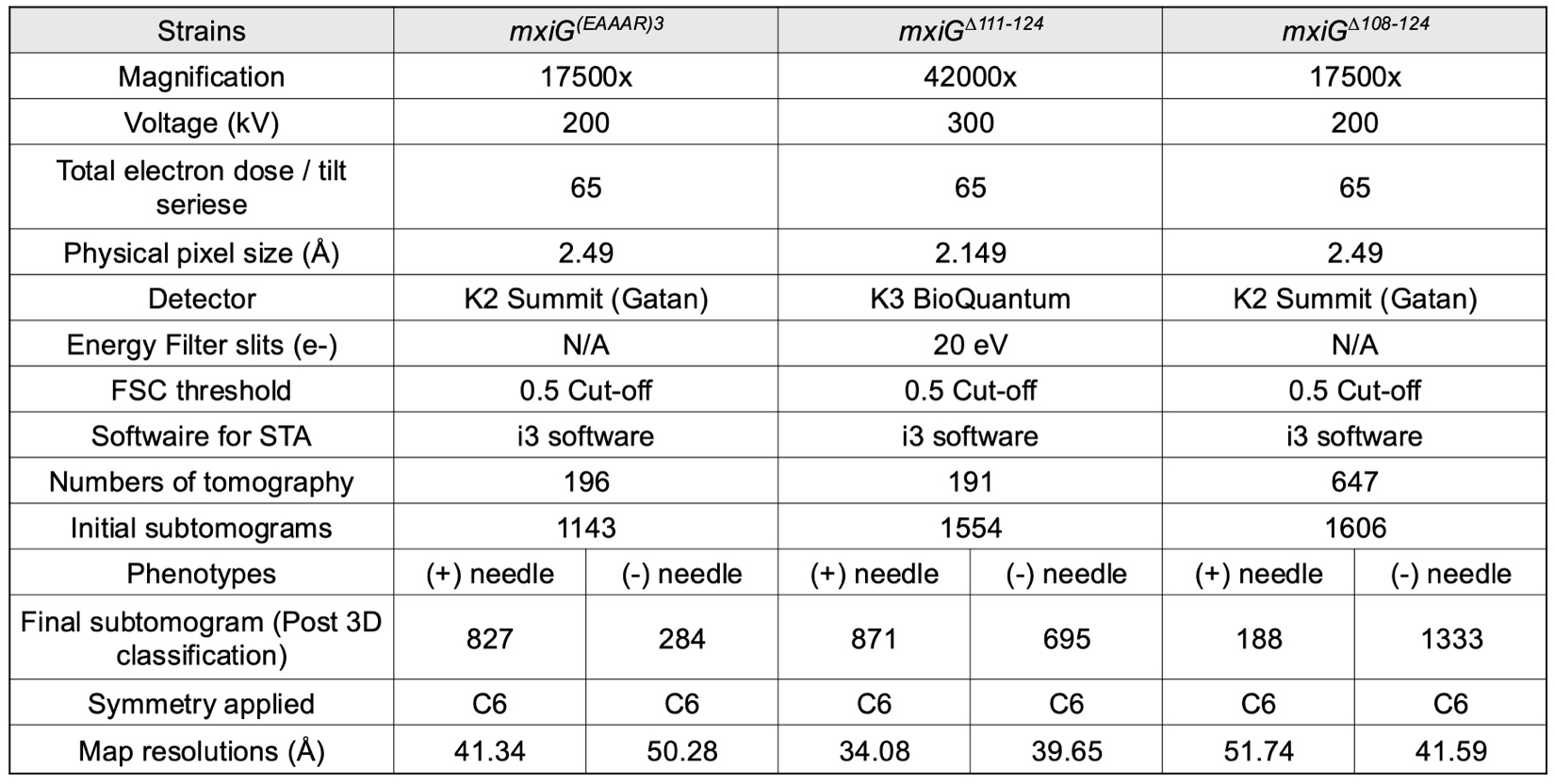


**Supplemental Table S3. Parameters of** cryo-ET data acquisition and data processing of the sub-tomogram averaging. Glacios (Thermofisher) was used for the data acquisition for the *mxiG^(EAAAR)3^* and *mxiG^∆108-124^* mutants, and Titan-Krios (Thermofisher) was used for the data acquisition for the *mxiG^∆111-124^* mutant.

**
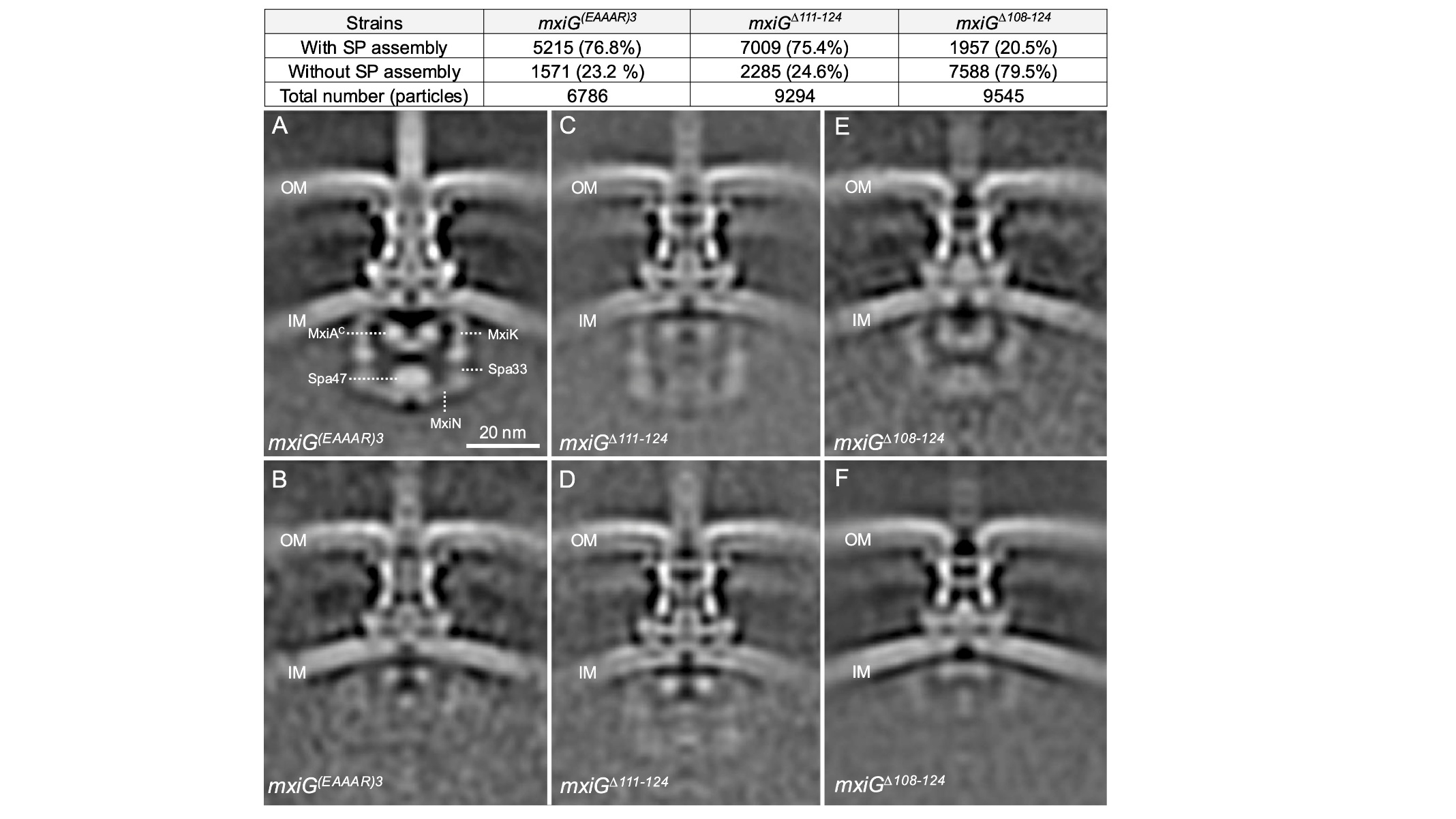
**

**Supplemental Figure S9.** The 3D classification was performed for the sorting platform (SP) region to classify injectisome with and without the SP in their structures. The **Top Panel** shows numbers of particles (injectisomes) in each class. **Panel A, C,** and **E** show averaged structure of injectisomes possessing the SP for MxiG^(EAAAR)3^, MxiG^∆111-124^, and MxiG^∆108-124^ mutant respectively. **Panel B, C,** and **E** show averaged structures of injectisome with less (or without) clear SP for each mutant.

**
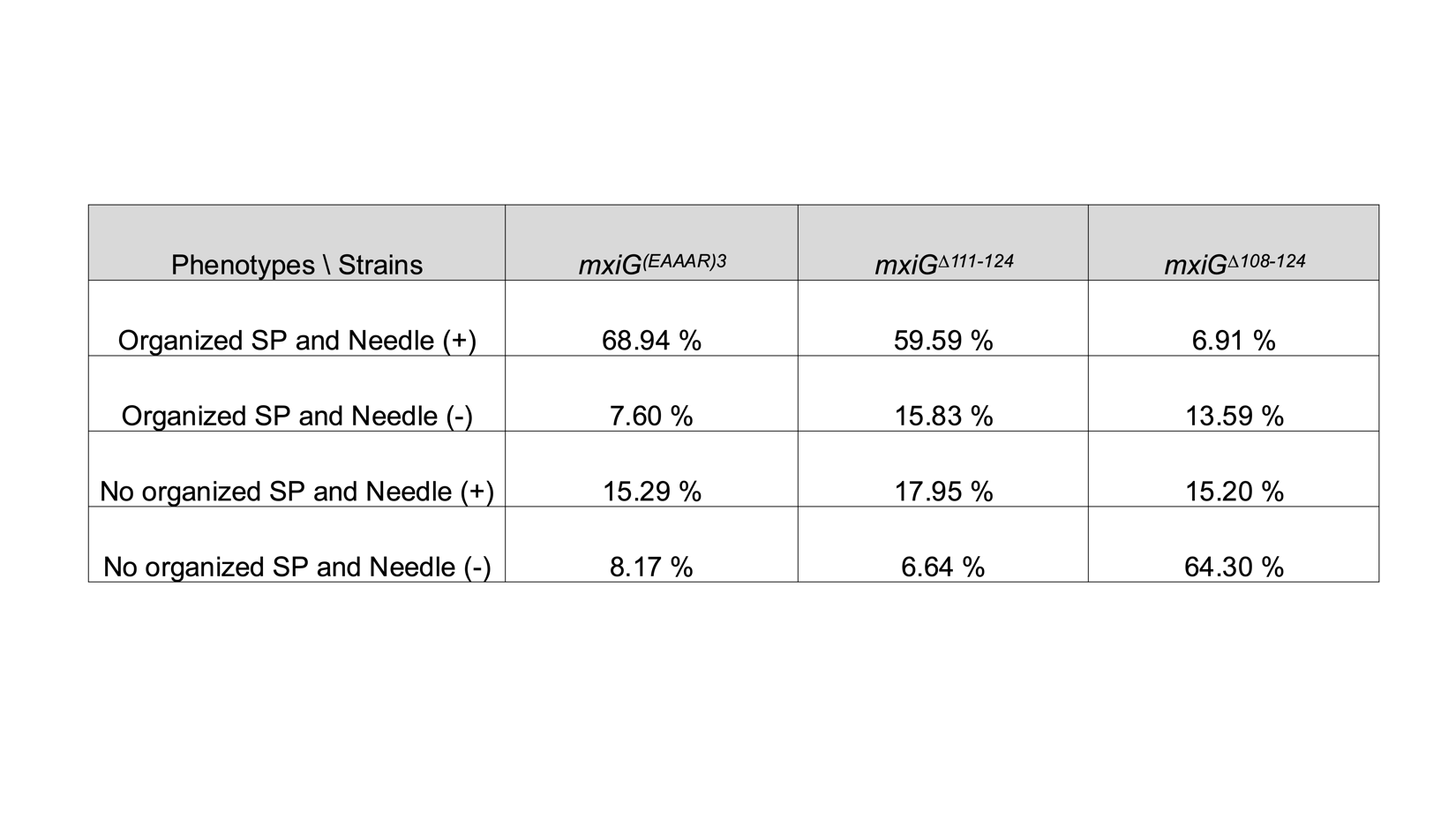
**

**Supplemental Table S4.** Frequency of double-positive, single-negative, and double-negative states of SP and needle density in individual injectisomes for the three MxiG linker mutants in Fig. 4.


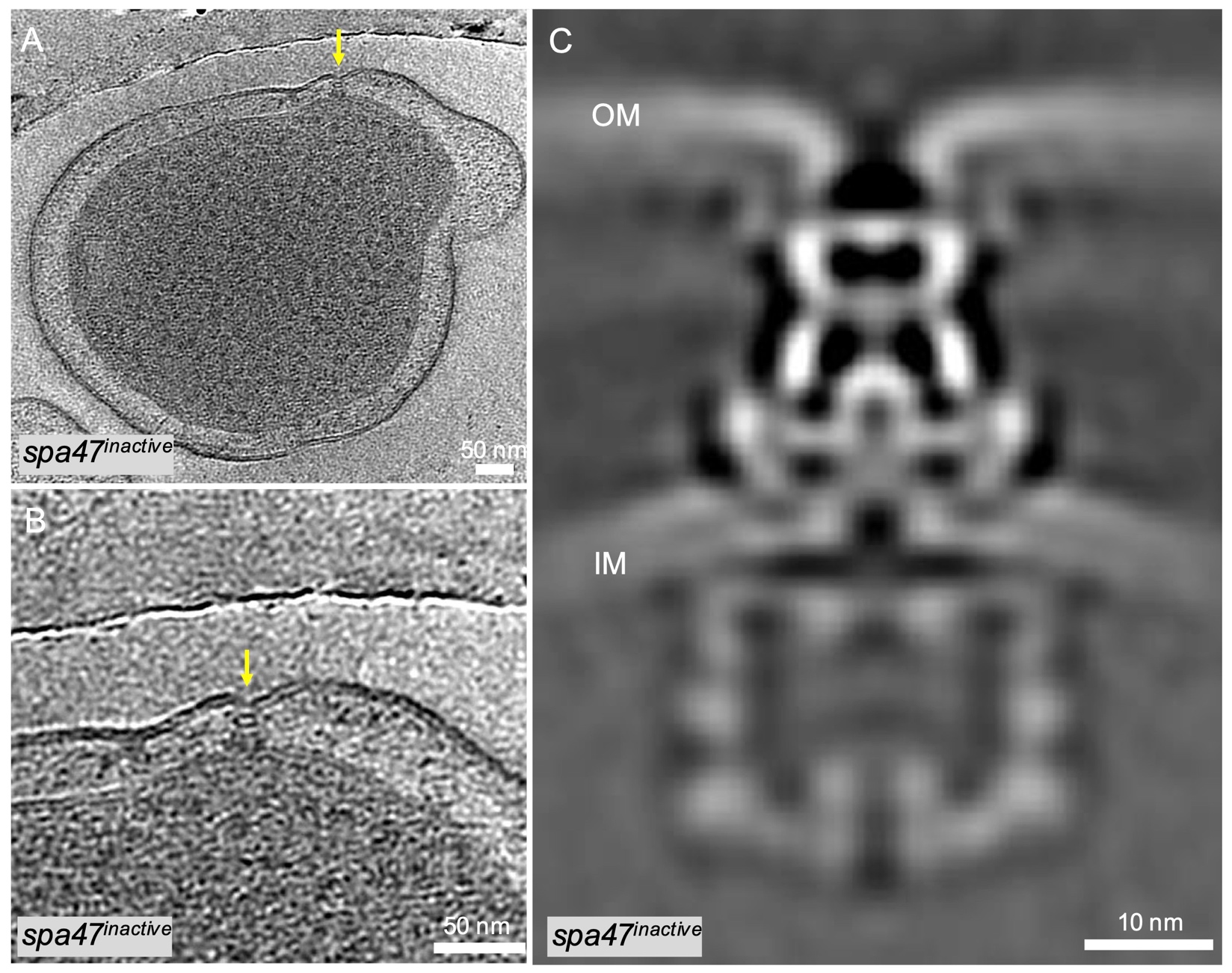


**Supplemental Figure S10.** **(A-B)** Cryo-ET imaging of *Shigella* minicells with the strain possessing a catalytically inactive form of the Spa47 ATPase. The panel B shows a zoomed in image of injectisome in the strain. There are no needles present on any of these injectisomes; however, an organized SP is present in the averaged structure **(C).**

| Primer/mutant Name | Direction | Sequence (5’- to -3’) | Primer Purpose |
| --- | --- | --- | --- |
| T4L Linker | Fw | GGTTCAATGAACATCTTCGAAATGCTGCGTATCGACGCTG | Amplify T4L for insertion into linker |
|  | Rev | TGAACCCCTGTAAGCGTCCCAGGTTCCG |  |
| N124-(Gly)_5_-H125 | Fw | GGTGGGGGAGGTGGGCACTCGGTGTTTTTTTTCTTTGCTGTTATT | Insert 5 Gly into MxiG linker |
|  | Rev | CCCACCTCCCCCACCGTTCTTATACATTCCGTTTAGTATATGGCCTCTCGA |  |
| N124-(Gly)_10_-H125 | Fw | GGTGGGGGAGGTGGGGGTGGGGGAGGTGGGCACTCGGTGTTTTTTTTCTT | Insert 10 Gly into MxiG linker |
|  | Rev | CCCACCTCCCCCACCCCCACCTCCCCCACCGTTCTTATACATTCCGTTTAGTATAT |  |
| ΔG115 and G120 | Fw | GAGGGAGGATAAATCGAGACATATACTAAACATGTATAAGAACCACTCGG | To clone MxiG^Δ115-120^ |
|  | Rev | CCGAGTGGTTCTTATACATGTTTAGTATATGTCTCGATTTATCCTCCCTC |  |
| ΔG115 to G120 | Fw | ATGTATAAGAACCACTCGGTGTTTTTTTTCTTTGC | To clone MxiG^Δ115-120^ |
|  | Rev | GTGGTTCTTATACATTCTCGATTTATCCTCCCTCATATTTTT |  |
| **ΔE110 to G120** | Rev | GTGGTTCTTATACATCCTCATATTTTTAAGGTGAAA | To clone MxiG^Δ110-120^ |
| **ΔM108 to G120** | Rev | GTGGTTCTTATACATATTTTTAAGGTGAAAAGAAATCCCGTC | To clone MxiG^Δ108-120^ |
| **ΔD111 to N124** | Rev | AAAAAACACCGAGTGCTCCCTCATATTTTTAAGG | To clone MxiG^Δ111-124^ |
| **ΔM108 to N124** | Fw | CACTCGGTGTTTTTTTTCTTTGCTGTTATTG | To clone MxiG^Δ108-124^ |
|  | Rev | AAAAAACACCGAGTGATTTTTAAGGTGAAAAGAAAT |  |
| G115-(T4L)-H116  (GFP) | Fw | GCTTACAGGGGTTCACATATACTAAACGGAATGTATAAG | To prepare insertion site at residue 115 in MxiG linker |
|  | Rev | GATGTTCATTGAACCGCCTCTCGATTTATCCTCCCT |  |
| **(Linker)_2_**  **2×Linker** | Fw | TTTCACCTTAAAAATATGAGAGAGGATAAA | To create two tandem linkers |
|  | Rev | AAAGAAAAAAAACACCGAATGGTT |  |
| **MxiG 127 Fw** | FW | GTGTTTTTTTTCTTTGCTGTTATTGTTGTGTTAATTATAATT | To prepare insertion site in MxiG for PrgH Linker and 2× Linker |
| PrgH Linker | Fw | ATTTCTTTTCACCTTAGCGAGCCGTGGGTGCCCGA | To amplify PrgH linker from *Salmonella* |
|  | Rev | AAAGAAAAAAAACACAATTCCGTTTTTAAAACGCGGCTCGTTCTTTTTTGCAGA |  |
| MxiG 105 Rev | Rev | AAGGTGAAAAGAAATCCCGTCATATTCAAA | To prepare insertion site in MxiG for PrgH |
| MxiG_107_Rev | Rev | ATTTTTAAGGTGAAAAGAAATCCCGTCATATTCAAA | To prepare insertion site in MxiG for 2× Linker |

**Supplemental Table S5. This is a list of the primers required to make the key MxiG mutants used in this study.** For additional mutants such as the poly-proline replacements and the (EAAAR)^n^ mutants, the entire linker region was generated with overlapping adjacent sequences for cloning into the parent MxiG.


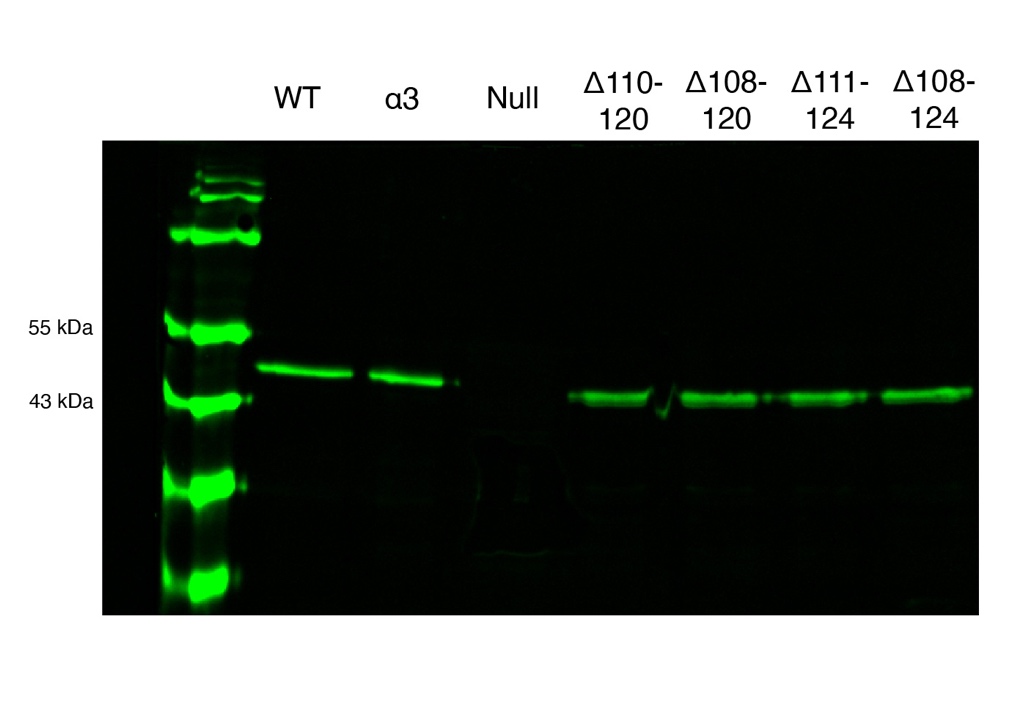


**Supplemental Figure S11.** Immunoblot analysis using anti-His6 antibodies recognizing a His6 tag on wildtype and the mutant forms of MxiG that were a focus of this study. The tagged MxiG was expressed in a *Shigella mxiG* null strain and the immunoblot shows the relative expression levels of each MxiG form expressed from pWPsf4. These data indicate that there are approximately equal levels of *mxiG* gene expression in the mutant strains and the sizes indicate that there is little or no degradation of the protein following its synthesis in *Shigella*. The expected molecular weight of the His6-tagged MxiG is ~ 47 kDa. The label α3 refers to the MxiG^(EAAAR)3^ mutant.
